# Redundant essentiality of AsmA-like proteins in *Pseudomonas aeruginosa*

**DOI:** 10.1128/msphere.00677-23

**Published:** 2024-02-02

**Authors:** Davide Sposato, Jessica Mercolino, Luisa Torrini, Paola Sperandeo, Massimiliano Lucidi, Riccardo Alegiani, Ilaria Varone, Giorgia Molesini, Livia Leoni, Giordano Rampioni, Paolo Visca, Francesco Imperi

**Affiliations:** 1 Department of Science, University Roma Tre, Rome, Italy; 2 Department of Pharmacological and Biomolecular Sciences, University of Milano, Milan, Italy; 3 NBFC, National Biodiversity Future Center, Palermo, Italy; 4 IRCCS Fondazione Santa Lucia, Rome, Italy; The University of Iowa, Iowa City, Iowa, USA

**Keywords:** * epaF*, glycerophospholipid transport, outer membrane, PA2708, PA4735, TamA, TamB, YhdP, YdbH

## Abstract

The outer membrane (OM) is an essential structure of Gram-negative bacteria that provides mechanical strength and protection from large and/or hydrophobic toxic molecules, including many antibiotics. The OM is composed of glycerophospholipids (GPLs) and lipopolysaccharide (LPS) in the inner and outer leaflets, respectively, and hosts integral β-barrel proteins and lipoproteins. While the systems responsible for translocation and insertion of LPS and OM proteins have been elucidated, the mechanism(s) mediating transport of GPLs from the inner membrane to the OM has remained elusive for decades. Very recently, studies performed in *Escherichia coli* proposed a role in this process for AsmA-like proteins that are predicted to share structural features with eukaryotic lipid transporters. In this study, we provide the first systematic investigation of AsmA-like proteins in a bacterium other than *E. coli*, the opportunistic human pathogen *Pseudomonas aeruginosa*. Bioinformatic analyses revealed that *P. aeruginosa* possesses seven AsmA-like proteins. Deletion of *asmA*-like genes in many different combinations, coupled with conditional mutagenesis, revealed that four AsmA-like proteins are redundantly essential for growth and OM integrity in *P. aeruginosa*, including a novel AsmA-like protein (PA4735) that is not present in *E. coli*. Cells depleted of AsmA-like proteins showed severe defects in the OM permeability barrier that were partially rescued by lowering the synthesis or transport of LPS. Since fine balancing of GPL and LPS levels is crucial for OM integrity, this evidence supports the role of AsmA-like proteins in GPL transport toward the OM.

**IMPORTANCE:**

Given the importance of the outer membrane (OM) for viability and antibiotic resistance in Gram-negative bacteria, in the last decades, several studies have focused on the characterization of the systems involved in OM biogenesis, which have also been explored as targets for antibacterial drug development. However, the mechanism mediating translocation of glycerophospholipids (GPLs) to the OM remained unknown until recent studies provided evidence that AsmA-like proteins could be responsible for this process. Here, we demonstrate for the first time that AsmA-like proteins are essential and redundant for growth and OM integrity in a Gram-negative bacterium other than the model organism *Escherichia coli* and demonstrate that the human pathogen *Pseudomonas aeruginosa* has an additional essential AsmA-like protein that is not present in *E. coli*, thus expanding the range of AsmA-like proteins that play key functions in Gram-negative bacteria.

## INTRODUCTION

The cell envelope of diderm (Gram-negative) bacteria is composed of two lipid bilayers, separated by an aqueous periplasmic space that contains a thin peptidoglycan cell wall (1). While the cytoplasmic inner membrane (IM) is a bilayer of glycerophospholipids (GPLs), the outer membrane (OM) is an asymmetric bilayer composed of GPLs in the inner leaflet and lipopolysaccharide (LPS) in the outer leaflet, which hosts integral β-barrel proteins (OMPs) and lipoproteins (2). The OM is an essential structure of Gram-negative bacteria, and the asymmetric assembly of its lipid components provides a selective permeability barrier that protects the cell from large and/or hydrophobic toxic molecules such as many antibiotics, partially accounting for the high intrinsic drug resistance of most Gram-negative pathogens (3). Recent studies uncovered that the OM also provides mechanical strength to compensate for the thin cell wall of Gram-negative bacteria (4).

Since the OM is essential and represents a major hurdle to antibiotic success against Gram-negative bacteria, the molecular machineries involved in its biogenesis have been investigated as targets for drug development (5–7). Interfering with OM biogenesis can directly kill bacterial cells or sensitize them to antimicrobials unable to cross a functional OM barrier (8, 9).

OM biogenesis is a complex process that poses several challenges. The biosynthesis of the major OM components takes place in the cytoplasm or at the IM and their transport to and assembly into the OM must be coordinated with cell growth and accomplished in an environment, the periplasm, that is devoid of obvious energy sources (1). Progress has been made in the past two decades in elucidating the molecular mechanisms that enable Gram-negative bacteria to overcome these challenges, leading to the discovery and characterization of dedicated protein machineries that mediate the assembly of individual OM components. OM proteins (OMPs and lipoproteins) are predominantly translocated across the IM in an unfolded state through the general secretory (Sec) system (10, 11). In the periplasm, OMPs are assisted by chaperones and delivered to the β-barrel assembly machinery (Bam), which promotes their final folding and integration into the OM (12, 13). Lipoproteins are maturated on the periplasmic side of the IM through the addition of the lipid moiety which anchors them to the IM. Then, OM lipoproteins are detached from the IM and shuttled to the OM by the Lol (localization of lipoproteins) system, which involves a soluble periplasmic protein (LolA) that masks the hydrophobic acyl chains from the aqueous periplasmic environment (14, 15). Intermembrane transport of LPS occurs through the seven-component LPS transport (Lpt) trans-envelope system, which forms a continuous protein bridge that connects the IM and OM and mediates LPS detachment from the IM, transit across the periplasm, and insertion into the outer leaflet of the OM (16, 17).

Despite this wealth of information on OM biogenesis, the mechanism mediating transport across the periplasm of the GPLs necessary for building the inner leaflet of the OM has remained elusive for decades. Several hypotheses have been formulated, including the presence of (i) hemifusion structures formed by the fusion of the IM and OM periplasmic leaflets into a contiguous bilayer crossing the periplasm, (ii) soluble periplasmic carriers, or (iii) proteinaceous tunnels connecting the IM and OM, but none of them has been experimentally confirmed yet (18, 19). This is mainly due to the inability to identify essential genes involved in this process, while it is logical to assume that GPL transport to the OM should be crucial for cell survival. In contrast, a system called Mla (maintenance of OM lipid asymmetry), that is devoted to maintaining OM asymmetry by removing mislocalized GPLs from the outer leaflet of the OM and transporting them back to the IM (retrograde transport), has been identified and characterized in several Gram-negative bacteria (18, 20, 21).

Recently, two independent studies performed in the model organism *Escherichia coli* demonstrated that three members of the AsmA-like protein clan (i.e., TamB, YhdP, and YdbH) are critical for OM integrity and proposed that they could provide a hydrophobic proteinaceous tunnel for GPL transport to the OM (22, 23). Indeed, single or double deletion mutants in these AsmA-like genes displayed impaired growth, cell morphology alterations, enhanced vesiculation, and/or increased antibiotic susceptibility, due to defects in OM integrity, while the triple deletion of *yhdP*, *tamB*, and *ydbH* was synthetically lethal, indicating that these genes are essential but redundant in *E. coli* (22, 23). Notably, *E. coli* cells impaired in the expression of these essential AsmA-like proteins were also found to accumulate GPLs in the IM (23), suggesting that these proteins are involved in GPL transport toward the OM. This is in line with the results of a previous study showing that the inactivation of the *yhdP* gene was able to reduce the flow of GPLs to the OM and, thus, attenuate the membrane defects of an *E. coli* mutant (mlaA*) in which the Mla system works in reverse, leading to GPL accumulation in the outer leaflet of the OM, increased flow of GPLs from the IM, and consequently, IM shrinkage (24). Notably, AsmA-like proteins are predicted to have an IM-anchoring N-terminal transmembrane helix (TMH) and a large periplasmic domain with a structure resembling that of the eukaryotic proteins (e.g., Vps13 and Atg2) involved in GPL transport between organelles (25–27).

Overall, this body of evidence strongly supports the role of AsmA-like proteins in GPL transport. However, up to date, the involvement of AsmA-like proteins in GPL transport and OM homeostasis has been systematically investigated only in *E. coli*. To provide further support to the possible role of AsmA-like proteins in the biogenesis of the Gram-negative cell envelope, we analyzed the effect of AsmA-like protein depletion in the opportunistic human pathogen *Pseudomonas aeruginosa*. Here, we confirm that AsmA-like proteins are essential and redundant for growth and OM integrity also in *P. aeruginosa* and provide indirect evidence that supports their role in the transport of GPLs to the OM. We also demonstrate that *P. aeruginosa* has an additional essential AsmA-like protein that is not present in *E. coli*, thus expanding the range of AsmA-like proteins that might play essential role(s) in diderm bacteria.

## RESULTS

### *P. aeruginosa* has seven AsmA-like proteins

The possible role of AsmA-like proteins in GPL transport and OM homeostasis has only been investigated in the model organism *E. coli*. This bacterium possesses six AsmA-like proteins (i.e., AsmA, TamB, YhdP, YdbH, YhjG, and YicH), of which TamB, YhdP, and YdbH were found to be essential but redundant for growth and OM integrity (22, 23).

**Fig 1:**
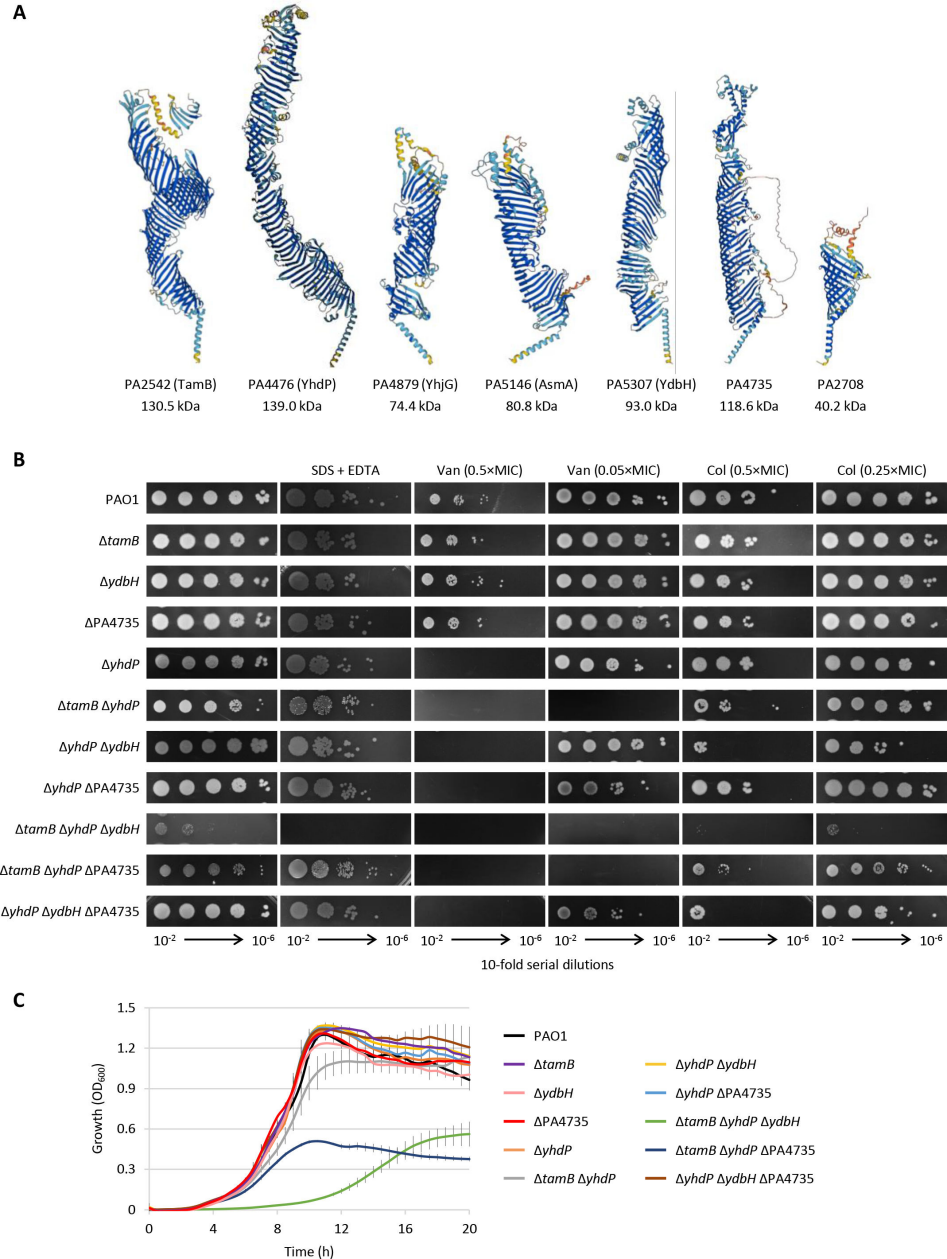
(**A**) Three-dimensional structures of *P. aeruginosa* AsmA-like proteins predicted by AlphaFold (https://alphafold.ebi.ac.uk/). The predicted molecular weights are also shown. (**B**) Plating efficiency of *P. aeruginosa* PAO1 and selected isogenic mutants with single, double, or triple deletions in *asmA*-like genes on MH agar plates supplemented or not with SDS and EDTA (0.25% and 0.25 mM, respectively), vancomycin (Van), or colistin (Col) at concentrations corresponding to 0.05, 0.25, or 0.5× MIC for the wild-type strain (as indicated). Images were taken after 24 h of incubation at 37°C and are representative of at least three independent experiments. The single mutants Δ*tamB*, Δ*ydbH*, and ΔPA4735 were included as controls. (**C**) Planktonic growth of *P. aeruginosa* PAO1 and selected isogenic mutants with single, double, or triple deletions in *asmA*-like genes in Mueller-Hinton (MH) broth in microtiter plates at 37°C. Values are the mean (±standard deviation) of three independent experiments performed in triplicate.

To verify the role of AsmA-like proteins in *P. aeruginosa*, we first searched for homologs of *E. coli* AsmA-like proteins in the *Pseudomonas* Genome Database (https://pseudomonas.com/) and for *P. aeruginosa* proteins in the Pfam database that belong to families within the AsmA-like clan. Seven genes encoding AsmA-like proteins were identified in the reference strain *P. aeruginosa* PAO1, all of which are conserved in the *P. aeruginosa* complete genomes deposited in the *Pseudomonas* Genome Database (Table 1). Five of these proteins have homology with *E. coli* proteins, and hereafter, they have been renamed as their putative *E. coli* orthologs, while a homolog of the *E. coli* protein YicH was not identified in *P. aeruginosa*. In addition, *P. aeruginosa* has two other AsmA-like proteins, PA4735 and PA2708, which are not present in *E. coli* (Table 1). The predicted three-dimensional structure of the *P. aeruginosa* AsmA-like proteins has been retrieved from the AlphaFold Protein Structure Database (https://alphafold.ebi.ac.uk/) (28). All *P. aeruginosa* AsmA-like proteins are predicted to share an N-terminal TMH and an elongated tube-like structure rich in β-strands with a lateral opening along the long axis (Fig. 1A). This structure resembles the periplasmic bridge made by Lpt proteins responsible for LPS transport across the periplasm (29, 30). Notably, the *P. aeruginosa* protein PA2708 is much smaller than the other AsmA-like proteins of both *P. aeruginosa* and *E. coli* (Table 1), and the length of its periplasmic domain is likely not sufficient to span the entire periplasmic space (Fig. 1A).

**TABLE 1:** AsmA-like proteins in *P. aeruginosa*

*P. aeruginosa* PAO1 AsmA-like protein (aa)	*E. coli* homolog (aa)	Identity (%)	Alignment length (aa)	E value	Pfam	% of *P. aeruginosa* genomes with ortholog^*a*^
PA2542 (1,221)	TamB (1,259)	25.81	1,259	0.0	PF04357	100^*b*^
PA4476 (1,276)	YhdP (1,266)	21.62	1,309	0.0	PF13502/PF13116	100^*c*^
PA4879 (688)	YhjG (686)	53.00	666	0.0	PF05170	100
PA5146 (750)	AsmA (617)	17.81	320	1.15E−6	PF05170	100
PA5307 (855)	YdbH (879)	24.49	147	7.13E−5	PF11739	100
PA4735 (1,088)	-^*e*^	-	-	-	PF05359	100
PA2708 (361)	-	-	-	-	PF05359	100^*d*^

^*a*^ Putative orthologs were retrieved from 257 complete genomes according to the *Pseudomonas* Ortholog Groups of the *Pseudomonas* Genome Database (https://pseudomonas.com/).

^*b*^ The ortholog of the *P. aeruginosa* strain PADK2_CF510 is probably a pseudogene.

^*c*^ The ortholog of the *P. aeruginosa* strain 2192 is probably a pseudogene.

^*d*^ The orthologs of the *P. aeruginosa* strains 2192; BWH034 and MRSN 20176 are probably pseudogenes.

^*e*^ The symbol "-" indicates the absence of a homolog in *E. coli*".

### Four AsmA-like proteins are redundantly essential in *P. aeruginosa*

In order to investigate the role of AsmA-like proteins in *P. aeruginosa*, we attempted to construct single, double, and triple in-frame deletion mutants in *asmA*-like genes in the reference strain *P. aeruginosa* PAO1. To reduce the number of possible combinations, at this stage, we decided to exclude PA2708 from the analysis, as its length and structure do not appear to be compatible with GPL transport across the cell envelope (Fig. 1A). However, the possible role of this protein has been investigated later in this study (see below). Forty-two mutants, corresponding to single, double, and triple mutants in *asmA*, *tamB*, *yhdP*, *ydbH*, *yhjG*, and PA4735 in all possible combinations, were generated (Table S1). Notably, although the concomitant inactivation of *tamB*, *yhdP*, and *ydbH* was found to be lethal in *E. coli* (22, 23), the triple mutant Δ*tamB* Δ*yhdP* Δ*ydbH* was successfully obtained in *P. aeruginosa*.

To preliminary screen all these mutants for defects in growth and/or OM integrity, we performed a qualitative spot plating assay on MH agar plates supplemented or not with the membrane-perturbing agents SDS and EDTA or sub-inhibitory concentrations of vancomycin (poorly active against Gram-negative cells with an intact OM) or colistin (that primarily targets the OM) (31–34). Several mutants displayed impaired growth and/or OM integrity defects with respect to the wild-type strain PAO1 (Fig. S1), thus confirming that AsmA-like proteins play important role(s) also in *P. aeruginosa*. The mutants that show an individual effect of each *asmA*-like gene on *P. aeruginosa* growth and OM integrity are shown in Fig. 1B. Single deletion of any *asmA*-like genes did not affect *P. aeruginosa* growth, even if the absence of YhdP made the cells more sensitive to vancomycin (no growth at 0.5× MIC for PAO1, corresponding to 1 mg/mL) (Fig. 1B; Fig. S1). The concomitant lack of YhdP and TamB (Δ*tamB* Δ*yhdP* mutant) slightly impaired growth (smaller colonies on MH agar) and hugely increased vancomycin sensitivity (no growth at 0.05× MIC for PAO1). The absence of YdbH in YhdP-deficient cells (Δ*yhdP* Δ*ydbH* mutant) slightly increased colistin sensitivity at both 0.5× and 0.25× MIC for PAO1 (corresponding to 0.25 and 0.125 µg/mL, respectively). Although viable, the triple mutant Δ*tamB* Δ*yhdP* Δ*ydbH* was strongly defective in growth and more sensitive to the membrane-destabilizing agents SDS and EDTA compared with the other strains (Fig. 1B). The deletion of *asmA* and/or *yhjG* in combination with all other mutations did not further impair growth under any conditions tested (Fig. S1), implying that these genes have no relevant effects on the phenotypes investigated in this assay. Interestingly, the inactivation of PA4735 in the Δ*tamB* Δ*yhdP* and Δ*yhdP* Δ*ydbH* mutants reduced the bacterial colony size under some conditions tested (Fig. 1B), suggesting that the lack of this protein might have a detrimental effect on *P. aeruginosa* cells defective in other important AsmA-like proteins.

To quantitatively assess the impact of AsmA-like proteins on *P. aeruginosa* growth, planktonic growth assays were performed in MH broth for all mutants. The growth curves of the single mutants (including Δ*yhdP*) and the wild-type strain were almost identical (Fig. 1C and data not shown), corroborating the results of the spot plating assay. A slight decrease in growth yields was observed for the double mutant Δ*tamB* Δ*yhdP* and, to a greater extent, for the triple mutant Δ*tamB* Δ*yhdP* ΔPA4735, while both growth rates and yields were drastically reduced in the triple mutant Δ*tamB* Δ*yhdP* Δ*ydbH* (Fig. 1C).

**Fig 2:**
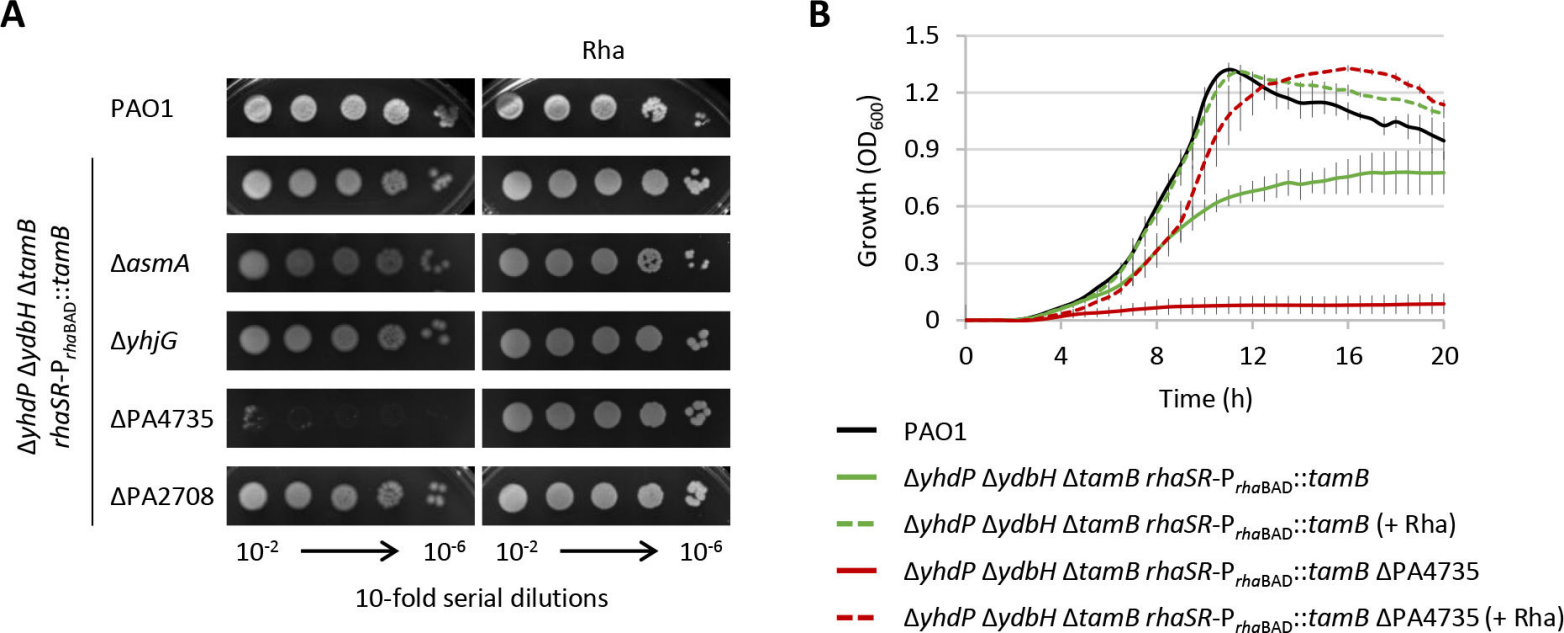
(**A**) Plating efficiency of *P. aeruginosa* PAO1, the rhamnose-dependent conditional mutant Δ*yhdP* Δ*ydbH* Δ*tamB rhaSR*-P*_rha_*_BAD_::*tamB*, and its derivatives deleted of *yhjG*, *asmA*, PA4735, or PA2708 on MH agar plates supplemented or not 0.01% rhamnose (Rha). Images were taken after 24 h of incubation at 37°C and are representative of three independent experiments. (**B**) Planktonic growth of the wild-type strain and the conditional mutants Δ*yhdP* Δ*ydbH* Δ*tamB rhaSR*-P*_rha_*_BAD_::*tamB* and Δ*yhdP* Δ*ydbH* Δ*tamB rhaSR*-P*_rha_*_BAD_::*tamB* ΔPA4735 in MH broth in microtiter plates at 37°C. Values are the mean (±standard deviation) of three independent experiments performed in triplicate.

These results demonstrate that YhdP, TamB, and YdbH are redundant and important for *P. aeruginosa* growth and OM integrity, in line with what was previously observed in *E. coli* (22, 23). However, the viability of the triple mutant Δ*tamB* Δ*yhdP* Δ*ydbH* in *P. aeruginosa* strongly suggests the presence of additional protein(s) in this bacterium that may be involved in the same physiological process, thus sustaining the low level of growth observed in the absence of YhdP, TamB, and YdbH. The detrimental effect of PA4735 inactivation on cells deficient in other AsmA-like proteins (Fig. 1) prompted us to investigate whether PA4735 could be responsible for the residual growth of Δ*tamB* Δ*yhdP* Δ*ydbH* cells. Several attempts to obtain a quadruple deletion mutant in *tamB*, *yhdP*, *ydbH*, and PA4735 failed, corroborating the hypothesis that PA4735 could be essential in YhdP-, TamB-, and YdbH-deficient cells. Therefore, we generated a conditional mutant carrying a rhamnose-dependent copy of *tamB* in a neutral site of the chromosome and deleted the endogenous copies of *tamB*, *yhdP*, and *ydbH* (Δ*yhdP* Δ*ydbH* Δ*tamB rhaSR*-P*_rhaBAD_::tamB*; Table S1). As expected, the growth of this conditional mutant was promoted by rhamnose (Fig. 2), although basal *tamB* expression in the absence of the inducer alleviated the growth defect relative to the triple-deletion mutant Δ*tamB* Δ*yhdP* Δ*ydbH* (Fig. 1 and 2). Then, *asmA*, *yhjG*, PA4735, and PA2708 genes were individually deleted in this conditional mutant. Growth assays on agar plates and in liquid medium revealed that *P. aeruginosa* growth was abrogated by the concomitant inactivation or depletion of YhdP, YdbH, TamB, and PA4735 (Fig. 2). In contrast, the deletion of *asmA*, *yhjG*, or PA2708 in the conditional mutant Δ*yhdP* Δ*ydbH* Δ*tamB rhaSR*-P*_rhaBAD_::tamB* did not exacerbate the growth defects of this strain under non-inducing conditions (Fig. 2A). This result demonstrates that PA2708, which was not investigated in the initial genetic screening, is not required for *P. aeruginosa* growth, and confirms that, compared with *E. coli*, *P. aeruginosa* has an additional AsmA-like protein (i.e., PA4735) that cooperates with YhdP, TamB, and YdbH to support bacterial growth.

**Fig 3:**
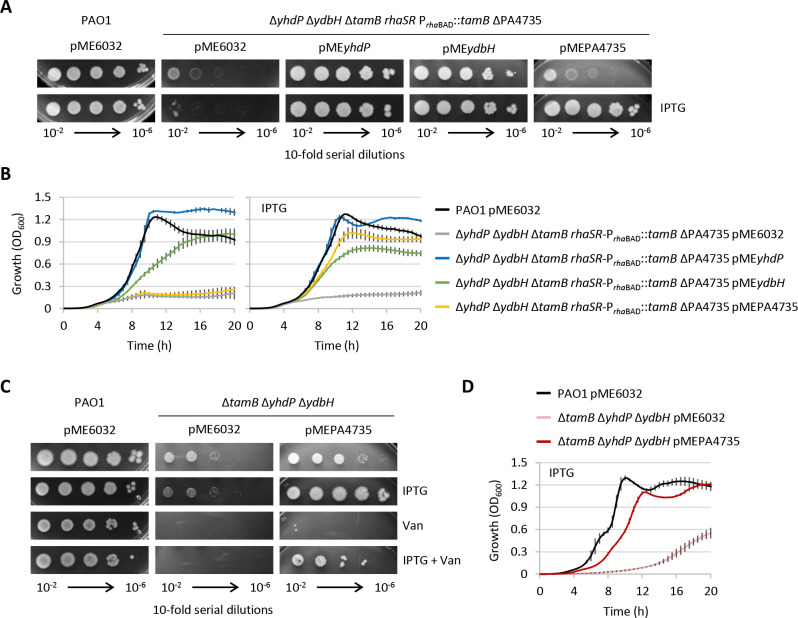
(**A**) Plating efficiency and (**B**) planktonic growth of the conditional mutant Δ*yhdP* Δ*ydbH* Δ*tamB rhaSR*-P*_rha_*_BAD_::*tamB* ΔPA4735 complemented with the IPTG-inducible construct pME*yhdP*, pME*ydbH*, or pMEPA4735 or the empty plasmid pME6032 as negative control, cultured in the presence or absence of 0.5 mM IPTG. (**C**) Plating efficiency and (**D**) planktonic growth of the triple mutant Δ*tamB* Δ*yhdP* Δ*ydbH* carrying pME6032 or pMEPA4735, cultured in the presence and/or absence of 0.5 mM IPTG and/or vancomycin at 100 µg/mL. In all experiments, *P. aeruginosa* PAO1 carrying pME6032 was used as the positive control of growth. Data are the mean (±standard deviation) or are representative of at least three independent experiments.

To further investigate this issue, we introduced a plasmid for the IPTG-inducible overexpression of PA4735 (pMEPA4735; Table S2) into the conditional mutant Δ*yhdP* Δ*ydbH* Δ*tamB rhaSR*-P*_rhaBAD_::tamB* ΔPA4735. For comparison, YhdP- or YdbH-expressing plasmids (i.e., pME*yhdP* and pME*ydbH*; Table S2) were also generated and tested. Plasmids carrying *yhdP* or *ydbH* were able to rescue the growth of the conditional mutant under non-permissive conditions (absence of rhamnose) both in the presence and in the absence of IPTG (Fig. 3A and B), indicating that the basal level of expression from the IPTG-dependent promoter in *P. aeruginosa* is sufficient to restore YhdP and YdbH functionality. Interestingly, ectopic expression of YdbH at both basal and induced levels did not restore growth at wild-type levels or at levels achieved upon YhdP (over)expression (Fig. 3B). This suggests that YdbH could be less effective than YhdP in performing the essential function(s) that AsmA-like proteins provide in *P. aeruginosa*. The introduction of pMEPA4735 in the conditional mutant did not restore growth unless IPTG was added to the medium (Fig. 3A and B), implying that high levels of PA4735 expression are required to support growth. This was confirmed by introducing the pMEPA4735 plasmid into the triple mutant Δ*tamB* Δ*yhdP* Δ*ydbH*, which expresses PA4735 at physiological levels. Upon induction with IPTG, the mutant carrying pMEPA4735 showed growth and vancomycin resistance profiles almost comparable to those of the wild-type strain (Fig. 3C and D), indicating that, when overexpressed, PA4735 is able to sustain growth and OM integrity even in the absence of the other essential AsmA-like proteins YhdP, TamB, and YdbH.

In conclusion, these results demonstrate that four AsmA-like proteins are essential but redundant in *P. aeruginosa*, including one (PA4735) that is not present in the model organism *E. coli*. A search for orthologs in the *Pseudomonas* Genome Database revealed that PA4735 is not restricted to *P. aeruginosa*, as putative PA4735 orthologs are present in many *Pseudomonas* species (https://pseudomonas.com/). While BLASTP search in the NCBI database did not identify any putative PA4735 homologs outside the *Pseudomonas* genus, structure-based comparisons in the AlphaFold database retrieved putative structural homologs of PA4735 in some distantly related bacteria belonging to different classes or phyla of diderm bacteria (Table S3). Alignment of the predicted 3D structures of PA4735 and some of these putative structural homologs confirmed that the overall fold of PA4735 is highly conserved in these proteins (Fig. S2). Even if this analysis is only based on structural predictions and might not be comprehensive, it strongly suggests that PA4735-like proteins are not exclusive to the *Pseudomonas* genus.

### AsmA-like proteins are important for cell morphology and membrane integrity

**Fig 4:**
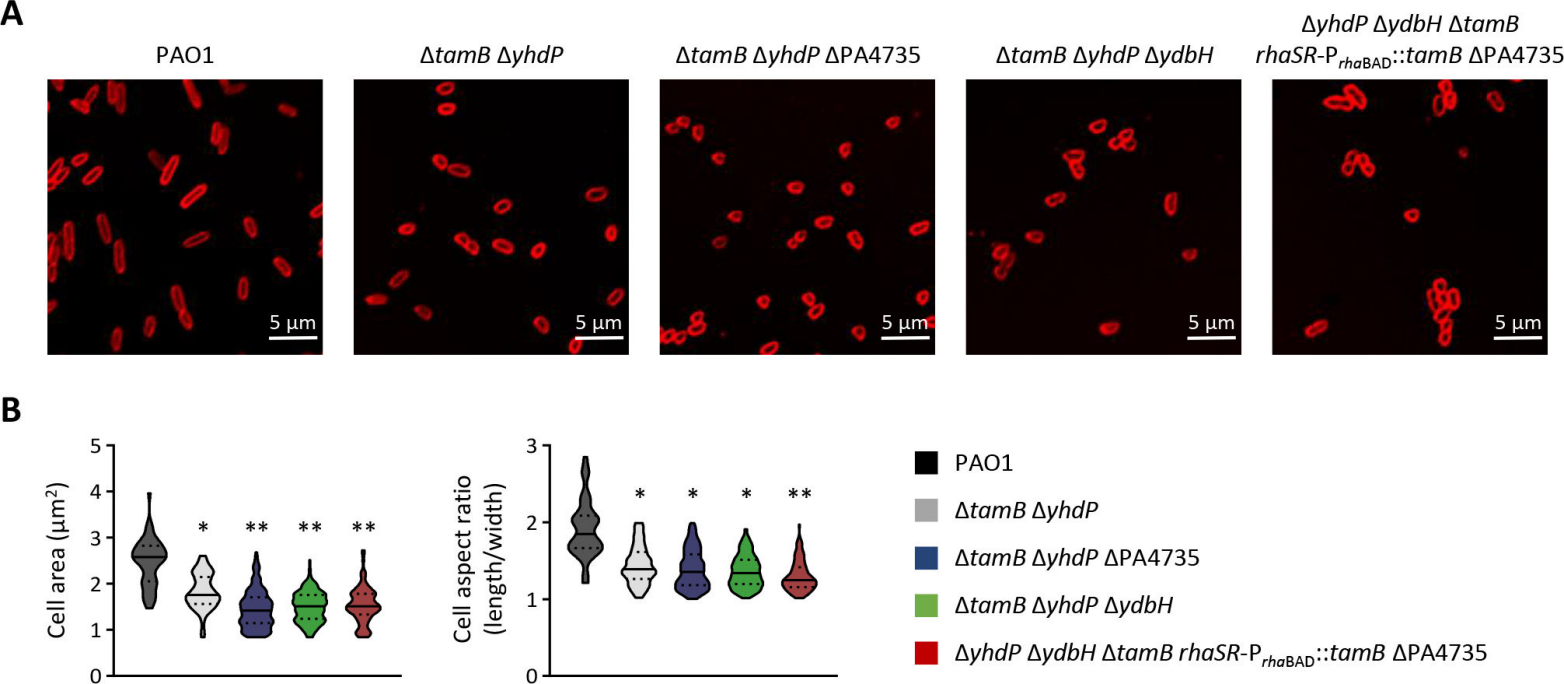
(**A**) Confocal microscopy images of *P. aeruginosa* PAO1 and the mutants Δ*tamB* Δ*yhdP*, Δ*tamB* Δ*yhdP* Δ*ydbH*, Δ*tamB* Δ*yhdP* ΔPA4735, and Δ*yhdP* Δ*ydbH* Δ*tamB rhaSR*-P*_rha_*_BAD_::*tamB* ΔPA4735 stained with the membrane-labeling dye FM4-64. Strains were cultured in MH until the mid-exponential phase, except for the conditional mutant Δ*yhdP* Δ*ydbH* Δ*tamB rhaSR*-P*_rha_*_BAD_::*tamB* ΔPA4735 that was progressively depleted of TamB using the culturing strategy shown in Fig. S4. Images are representative of two independent experiments and several fields of view showing the same results. (**B**) Violin plots showing cell area (μm^2^) and aspect ratio (length/width) for 100 cells of the same strains shown in panel A, calculated with the ImageJ software. Asterisks indicate a statistically significant difference with respect to the wild-type strain (*P < 0.05 and **P < 0.01; unpaired t test). Confocal microscopy images and analysis of the single mutants Δ*tamB*, Δ*yhdP*, Δ*ydbH*, and ΔPA4735 are shown in Fig. S3.

In *E. coli*, depletion of essential AsmA-like proteins causes significant defects in cell morphology and membrane permeability (22, 23). Confocal microscopy imaging of cells stained with the membrane-labeling dye FM4-64 showed that cells of the double mutant Δ*tamB* Δ*yhdP* are smaller (reduced cell area) and rounder (lower length/width ratio) than wild-type cells (Fig. 4 and S3), while all other single or double mutants did not show any apparent alteration of cell morphology (data not shown). Comparable phenotypes were observed for the triple mutants Δ*tamB* Δ*yhdP* ΔPA4735 and Δ*tamB* Δ*yhdP* Δ*ydbH*, as well as for cells of the conditional mutant Δ*yhdP* Δ*ydbH* Δ*tamB rhaSR*-P*_rhaBAD_::tamB* ΔPA4735, whose growth was arrested by progressively reducing the concentration of rhamnose in the medium (Fig. 4 and S4). This suggests that the lack of TamB and YhdP proteins is mainly responsible for the observed cell elongation defects, even if growth is only marginally affected in TamB- and YhdP-depleted cells (Fig. 1). Notably, under non-permissive conditions, the growth of the conditional mutant Δ*yhdP* Δ*ydbH* Δ*tamB rhaSR*-P*_rhaBAD_::tamB* ΔPA4735 was arrested without a relevant decrease in the optical density at 600 nm (OD_600_), suggesting that depletion of essential AsmA-like proteins does not cause lysis in *P. aeruginosa*, different from what was observed in *E. coli* (22).

The introduction of a plasmid expressing an FtsZ-GFP fusion protein (pME*ftsZ-GFP*; Table S2) (35) allowed demonstrating that the Z-ring is assembled and correctly localized at mid-cell in double and triple *asmA*-like gene mutants (Fig. S5), leading to hypothesizing that depletion of essential AsmA-like proteins does not directly affect the divisome assembly and, plausibly, cell constriction.

**Fig 5:**
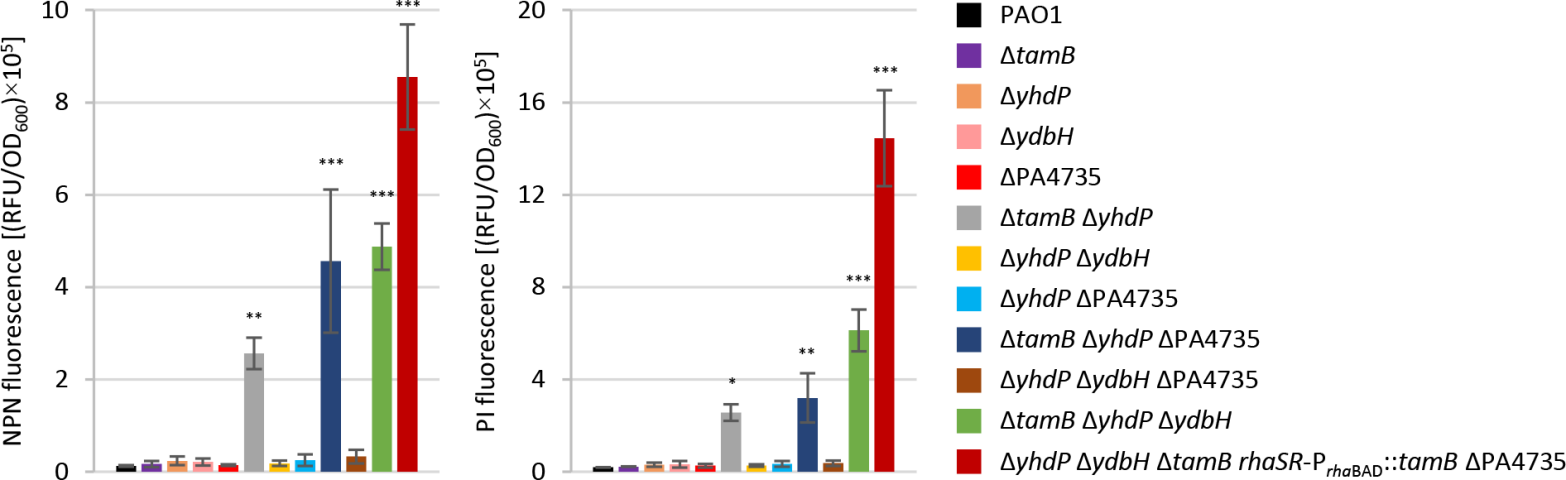
Uptake of NPN (left panel) and PI (right panel) by *P. aeruginosa* PAO1 and selected isogenic mutants, measured as relative fluorescence units (RFU) normalized to the OD_600_ of the bacterial suspension. Strains were cultured in MH until the mid-exponential phase, except for the conditional mutant Δ*yhdP* Δ*ydbH* Δ*tamB rhaSR*-P*_rha_*_BAD_::*tamB* ΔPA4735 that was progressively depleted of TamB using the culturing strategy shown in Fig. S4. Values are the mean (±standard deviation) of three independent experiments, and asterisks indicate a statistically significant difference with respect to the wild-type strain (*P < 0.05, **P < 0.01, and ***P < 0.001; ANOVA).

The importance of AsmA-like proteins for membrane functionality was investigated through cell permeability assays with the fluorescent probes 1-N-phenylnaphthylamine (NPN) and propidium iodide (PI), on strains that showed growth or antibiotic resistance defects (Fig. 1 and 2). NPN is a hydrophobic probe whose fluorescence is strongly increased upon binding to membrane lipids and is commonly used to measure OM destabilization, while PI only enters cells with damaged membranes and increases its fluorescence upon binding to nucleic acids (36, 37). Cells lacking TamB and YhdP were found to be 20-fold more permeable to NPN than wild-type cells (Fig. 5), indicating that the permeability barrier of the OM is significantly compromised. Permeability to NPN was further increased in TamB- and YhdP-deficient cells also lacking PA4735 or YdbH (Fig. 5), indicating that all these AsmA-like proteins contribute to some extent to OM homeostasis. Accordingly, the OM of *P. aeruginosa* cells was extremely permeable to NPN when the four AsmA-like proteins were concomitantly depleted (conditional mutant Δ*yhdP* Δ*ydbH* Δ*tamB rhaSR*-P*_rhaBAD_::tamB* ΔPA4735 in Fig. 5). The PI permeability profiles of all these mutants substantially mirrored those of NPN (Fig. 5), confirming that AsmA-like protein depletion causes relevant defects in membrane functionality.

### Lowering LPS synthesis or transport partially rescues the OM defects caused by AsmA-like protein depletion

Reduced biosynthesis or transport of GPLs is expected to affect bacterial cell size and growth rate by hampering the ability of the bacterium to create biomass for cell elongation and division (38, 39). However, the integrity and functionality of the OM as a permeability barrier mainly rely on the balance between the LPS and GPL content in the outer and inner leaflets, respectively (23, 38, 40, 41). Thus, if AsmA-like proteins are indeed responsible for GPL transport from the IM to the OM, one might expect the OM defects of cells depleted of AsmA-like proteins to be rescued by reducing the amount of LPS in the OM. This has been demonstrated in *E. coli*, where the replacement of the LPS biosynthetic gene *lpxC* with the less functional allele *lpxC101*, resulting in reduced LPS synthesis, partially restored OM functionality in cells deficient in essential AsmA-like proteins (23). We attempted to demonstrate if the same occurs in *P. aeruginosa* by using two complementary approaches, based on (i) chemical inhibition of LpxC activity or (ii) conditional mutagenesis to lower the expression of the *lptB* gene, which encodes the ATPase essential to energize LPS transport through the Lpt machinery (42).

**Fig 6:**
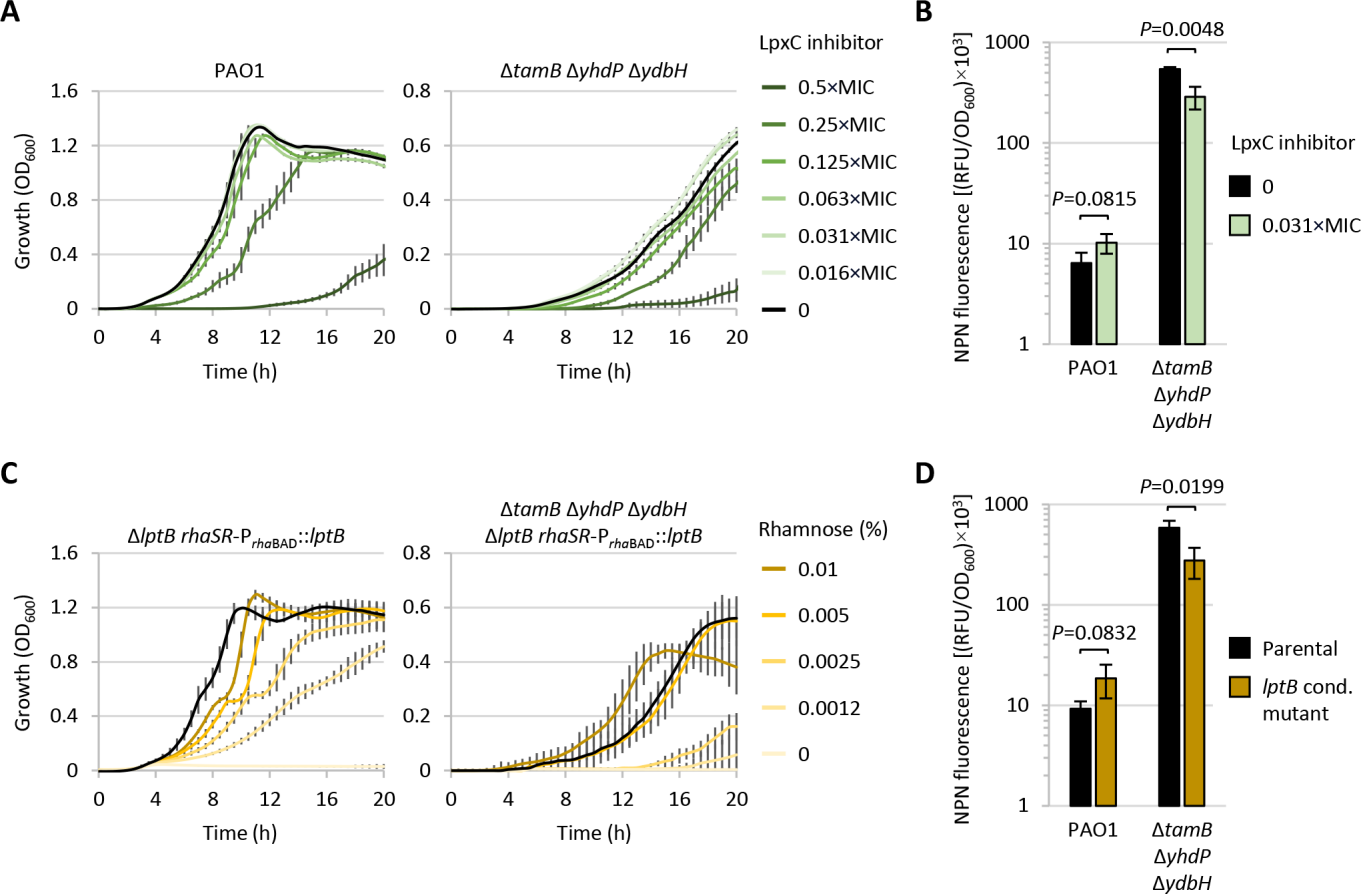
(**A**) Planktonic growth of *P. aeruginosa* PAO1 and the triple mutant Δ*tamB* Δ*yhdP* Δ*ydbH* cultured in MH broth in the presence or absence of different sub-inhibitory concentrations of the LpxC inhibitor PF-5081090. The MIC of PF-5081090 was 0.25 µg/mL for PAO1 and 0.125 µg/mL for Δ*tamB* Δ*yhdP* Δ*ydbH*. (**B**) Uptake of NPN by the same strains described in panel A cultured until mid-exponential phase in the presence or absence of 0.031× MIC of PF-5081090, measured as RFU normalized to the OD_600_ of the bacterial suspension. (**C**) Planktonic growth of the conditional mutants Δ*lptB rhaSR*-P*_rha_*_BAD_::*lptB* and Δ*tamB* Δ*yhdP* Δ*ydbH* Δ*lptB rhaSR*-P*_rha_*_BAD_::*lptB* cultured in MH broth in the presence or absence of different concentrations of rhamnose as indicated. The parental strains PAO1 (left panel) and Δ*tamB* Δ*yhdP* Δ*ydbH* (right panel) cultured in the absence of rhamnose were included as controls (black lines). (**D**) Uptake of NPN by the same strains described in panel C cultured until mid-exponential phase in the presence of rhamnose at 0.01% (Δ*lptB rhaSR*-P*_rha_*_BAD_::*lptB*) or 0.005% (Δ*tamB* Δ*yhdP* Δ*ydbH* Δ*lptB rhaSR*-P*_rha_*_BAD_::*lptB*), measured as RFU normalized to the OD_600_ of the bacterial suspension. Values are the mean (±standard deviation) of three independent experiments. The unpaired t test was used to assess statistically significant differences between treated and untreated samples in panel B and between parental strains and the cognate *lptB* conditional mutants in panel D.

For chemical inhibition, we used the LpxC inhibitor PF-5081090 previously demonstrated to be active against many Gram-negative bacteria including *P. aeruginosa* (43). Preliminary growth assays in the presence of different concentrations of PF-5081090 were performed for the wild-type strain PAO1 and the triple mutant Δ*tamB* Δ*yhdP* Δ*ydbH* to select the highest concentration that did not affect growth (Fig. 6A). Then, strains were cultured in the presence of the selected sub-inhibitory PF-5081090 concentration (0.031× MIC) and the NPN permeability assay was performed to monitor OM functionality. In wild-type cells, PF-5081090 treatment caused a mild (1.6-fold) increase in OM permeability (Fig. 6B), in line with what was previously observed in *P. aeruginosa* cells impaired in LPS transport (44). Conversely, treatment with an equivalent amount of PF-5081090 led to a significant twofold reduction of OM permeability in the Δ*tamB* Δ*yhdP* Δ*ydbH* mutant (Fig. 6B), though this was not sufficient to restore growth and OM integrity at wild-type levels (Fig. 6A and B)

Almost identical results were obtained by placing the LPS transport gene *lptB* under the control of a rhamnose-dependent promoter in PAO1 and in the Δ*tamB* Δ*yhdP* Δ*ydbH* mutant. As expected, the growth of both conditional mutants (Δ*lptB rhaSR*-P*_rha_*_BAD_::*lptB* and Δ*tamB* Δ*yhdP* Δ*ydbH* Δ*lptB rhaSR*-P*_rha_*_BAD_::*lptB*; Table S1) was promoted by rhamnose in a dose-dependent manner (Fig. 6C), confirming the essentiality of the Lpt system in *P. aeruginosa* (45). By performing the NPN permeability assay on cells cultured in the presence of sub-optimal concentrations of rhamnose, again, we observed that the OM was twofold more permeable in the AsmA-like protein-proficient strain and twofold less permeable in the strain deficient in AsmA-like proteins (Fig. 6D).

Overall, these results demonstrate that the OM of cells defective in AsmA-like protein function(s) can be partially stabilized by reducing LPS levels in the OM, thus corroborating the proposed role of AsmA-like proteins in GPL transport to the OM (22–24, 26).

### TamA is only required for TamB activity

**Fig 7:**
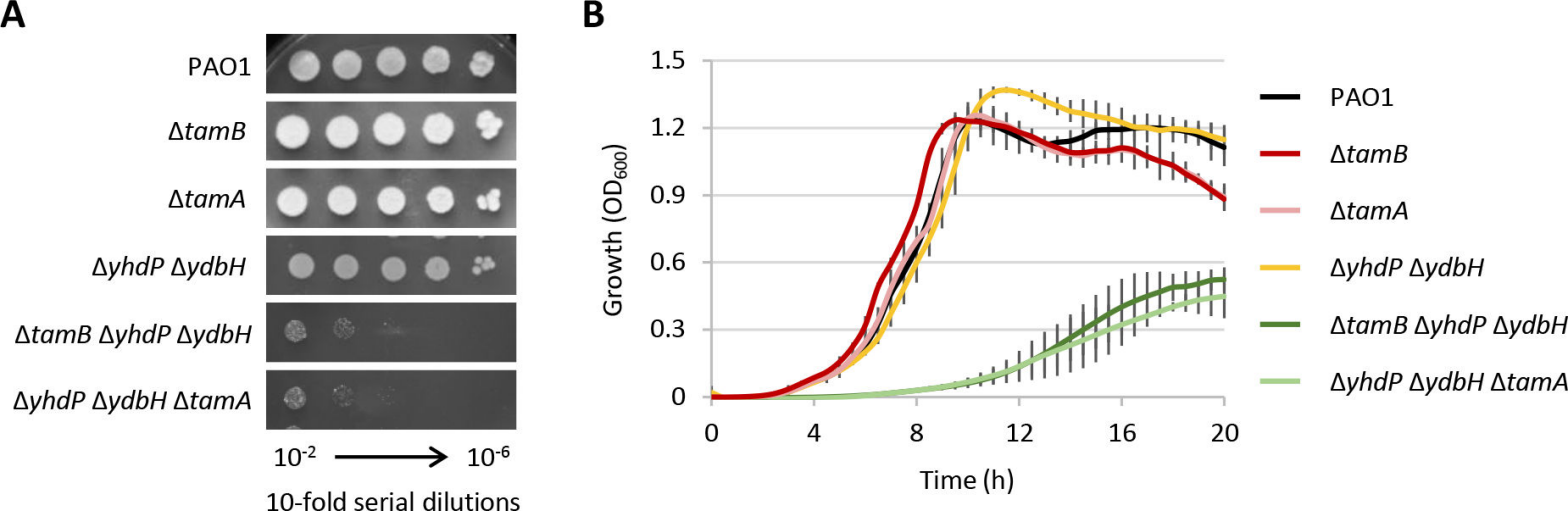
Comparison of (**A**) plating efficiency and (**B**) planktonic growth between selected *tamA* and *tamB* mutants on MH agar plates or in MH broth. The wild-type strain PAO1 and the double mutant Δ*yhdP* Δ*ydbH* were used as controls. Data are the mean (±standard deviation) or are representative of at least three independent experiments.

In *E. coli*, TamB interacts with the OM β-barrel protein TamA to form the so-called TAM complex (46), which was originally proposed to be involved in the insertion into the OM of a subset of β-barrel proteins, such as autotransporters and fimbrial ushers (47, 48). More recently, genetic evidence highlighted that TamA is required for the function of TamB in maintaining OM homeostasis in *E. coli* (22). Since a *tamA* ortholog is present in *P. aeruginosa* (PA2543 in PAO1) and appears to be operonic with *tamB* (https://pseudomonas.com/), we decided to verify whether TamA is required for TamB activity also in this bacterium. To this aim, we generated an in-frame deletion in *tamA* in the wild-type and Δ*yhdP* Δ*ydbH* backgrounds. The single mutant Δ*tamA* did not show any growth defect, in line with what was observed for the Δ*tamB* mutant (Fig. 7). In contrast, deletion of *tamA* in the mutant lacking YhdP and YdbH drastically reduced growth, both on agar plates and in liquid medium. Notably, the growth profile of the Δ*yhdP* Δ*ydbH* Δ*tamA* mutant was almost identical to that of the Δ*tamB* Δ*yhdP* Δ*ydbH* mutant (Fig. 7), strongly suggesting that the absence of TamA makes TamB inactive and, thus, that TamB-TamA interaction is likely important for TamB functionality also in *P. aeruginosa*. Notably, the Δ*tamA* mutant also showed no increase in NPN permeability or vancomycin sensitivity (data not shown), indicating that the integrity of the OM is not (or only marginally) compromised in TamA-deficient *P. aeruginosa* cells. Since OM integrity is very sensitive to the absence of YhdP, YdbH, and/or PA4735 (Fig. 1 and 5), this finding leads us to propose that the other essential AsmA-like proteins do not rely on TamA for their function(s).

## DISCUSSION

The anterograde transport of GPLs from the IM to the OM of Gram-negative bacteria is one of the most elusive essential physiological processes in bacteria. Recently, independent studies performed in the model organism *E. coli* provided genetic and biochemical evidence that proteins belonging to the AsmA-like clan could account for this task (22–24). Although a direct demonstration that AsmA-like proteins are able to bind and transport GPLs still lacks, it has been shown that *E. coli* cells depleted of the AsmA-like proteins YhdP, TamB, and/or YdbH (i) have a reduced flux of GPLs toward the OM (24); (ii) are defective in growth, cell elongation, OM integrity, and antibiotic resistance, all phenotypes reminiscent of impaired OM biogenesis (22, 23); and (iii) accumulate GPLs in the IM (23). These findings, together with the predicted subcellular localization and tube-like structure of AsmA-like proteins, resembling eukaryotic inter-organelle GPL transporters and Lpt proteins responsible for LPS transport toward the OM (26, 27, 29, 30), strongly support the hypothesis that these proteins may represent the long sought-after mechanism for GPL translocation from the IM to the OM (22, 23, 26).

Since GPL transport is expected to be essential for OM biogenesis in all diderm bacteria, proving that AsmA-like proteins play a key role in cell envelope biogenesis and cell viability also in other species is pivotal to propose these proteins as the universal GPL transport mechanism of Gram-negative bacteria. Although the possible role of AsmA-like proteins in GPL transport has only been recently proposed (22, 23), the effect of the lack of specific AsmA-like protein(s) has been occasionally investigated in diderm bacteria other than *E. coli*. Most of the studies were focused on TamB orthologs, probably because of the originally proposed role of this AsmA-like protein in the maturation of a subset of OM β-barrel proteins (46–48). Similar to what was observed in *E. coli*, the *Brucella suis* TamB homolog MapB is required for proper assembly of an autotransporter adhesin in the OM, although MapB-deficient cells also display anomalies in cell morphology and division (49). The lack of the TamB homolog MorC in *Aggregatibacter actinomycetemcomitans* does not affect autotransporter levels in the OM (50) but changes OM morphology from rugose to flat, decreases cell size, and induces autoaggregation (51). Likewise, the TamB homolog DR_146T of *Deinococcus radiodurans* is important for the maintenance of cell envelope integrity and for resistance to shear and osmotic stresses (52), while the lack of TamB in the intracellular pathogen *Edwardsiella tarda* leads to pleiotropic defects in membrane integrity under stresses, motility, cell invasion, and intracellular replication (53). In *Salmonella*, the Tam system does not appear to be involved in the assembly of autotransporters but affects OM homeostasis and shows some functional interplay with the Bam machinery (54). Finally, it has been recently found that *tamB* inactivation alleviates the OM lipid asymmetry defects of a Mla-defective mutant of *Veillonella parvula*, a diderm member of the Gram-positive phylum Firmicutes (55), suggesting that the role of TamB in OM lipid homeostasis may be widely conserved.

In this study, we provide the first systematic investigation of AsmA-like proteins in a Gram-negative bacterium different from *E. coli*. Our results revealed that, in line with what was previously demonstrated in *E. coli*, *P. aeruginosa* has multiple highly conserved AsmA-like proteins, some of which are essential but redundant for growth and OM integrity. The three essential AsmA-like proteins of *E. coli* (i.e., YhdP, TamB, and YdbH) seem to be functionally conserved in *P. aeruginosa*, as well as their relative contribution to OM homeostasis, with YhdP playing a more critical role than TamB and YdbH (Fig. 1) (22). However, we also found that an additional AsmA-like protein absent in *E. coli*, PA4735, compensates for the lack of YhdP, TamB, and YdbH, thus supporting the growth of the triple mutant Δ*tamB* Δ*yhdP* Δ*ydbH* in *P. aeruginosa* (Fig. 2 and 3). We therefore propose to name the PA4735 gene *epaF* (for essential *Pseudomonas* AsmA-like protein four). While sequence homology search suggested that this protein may be specific to the *Pseudomonas* genes, we identified putative structural homologs in several diderm bacteria (Table S3 and Fig. S2), implying that this protein might be involved in cell envelope biogenesis also in other species. Although we did not provide direct evidence of GPL translocation through AsmA-like proteins, we confirmed that also in *P. aeruginosa*, the defects in the OM permeability barrier caused by AsmA-like protein deficiency can be partially rescued by reducing the synthesis or transport of LPS (Fig. 6), as previously observed in *E. coli* (23). This is consistent with the notion that the balance between LPS and GPL in the OM is crucial for maintaining OM asymmetry and a functional permeability barrier (23, 38, 40, 41), thus indirectly supporting the proposed role of AsmA-like proteins in GPL anterograde transport (22, 23, 26).

The *P. aeruginosa* genomes have three other *asmA*-like genes that, based on the assays performed in this study, do not seem to play a major role in OM biogenesis or homeostasis. Two of them encode putative homologs of the *E. coli* proteins AsmA and YhjG. While inactivation of *yhjG*, alone or in combination with other *asmA*-like genes, has no or very low impact on phenotypes related to OM integrity also in *E. coli* (22, 23), the effect of AsmA deficiency on *P. aeruginosa* markedly differs from that observed in the model organism *E. coli*. Indeed, the importance of AsmA for *E. coli* cell envelope homeostasis has been known for years (56) and has been recently confirmed (22). In contrast, we did not observe any effects of *asmA* deletion on *P. aeruginosa*, even when combined with the inactivation of multiple *asmA*-like genes (Fig. S1 and S2). Notably, the *P. aeruginosa* and *E. coli* AsmA proteins are highly divergent in terms of sequence homology and size, with the *P. aeruginosa* protein being 20% longer than the *E. coli* counterpart (Table 1), suggesting that either they are not actually orthologs or they diverged toward different functions in these two bacteria. However, the possibility that *asmA* in *P. aeruginosa* is only expressed in response to specific stimuli cannot be ruled out. The third non-essential AsmA-like protein of *P. aeruginosa* (PA2708) is much shorter than all the other AsmA-like proteins characterized so far (Table 1 and Fig. 1) (22, 23, 49–55), and its size is not expected to allow to cross the periplasmic space unless PA2708 can interact with OM partners protruding into the periplasm. Interestingly, this protein is conserved in different *Pseudomonas* species (https://pseudomonas.com/), indicating that it has been maintained during *Pseudomonas* evolution. Further studies are required to uncover its role in *Pseudomonas* physiology.

Overall, this work supports the recently proposed role of AsmA-like proteins in GPL transport, by demonstrating that a subset of AsmA-like proteins is redundantly essential for growth and OM biogenesis also in *P. aeruginosa*, and provides evidence that the repertoire of essential AsmA-like proteins of Gram-negative bacteria might be more variegated than that described in the model organism *E. coli*. However, there are still many open issues that need to be addressed to confirm the involvement of AsmA-like proteins in GPL anterograde transport and to decipher their mechanism of action. First, there is no biochemical and structural evidence that confirms the capability of AsmA-like proteins to interact with GPLs. Second, no data are available on the mechanism(s) by which AsmA-like proteins could extract GPLs from the IM and insert them into the OM. The OM β-barrel protein TamA is required for TamB function in OM biogenesis, both in *E. coli* and *P. aeruginosa* (22) (Fig. 7). Moreover, TamA possesses a lateral gate in the barrel domain similar to the one present in BamA that allows insertion of OM β-barrel proteins (57, 58). Thus, TamA could provide a possible route for inserting GPLs into the OM. However, since TamA is generally not essential in Gram-negative bacteria (59), it is unlikely that TamA represents a universal OM partner of AsmA-like proteins for GPL insertion into the OM. This would also be in line with phylogenomic evidence suggesting that many diderm bacteria have TamB but not TamA orthologs (60). Thus, either the other OM partner(s) of AsmA-like proteins have yet to be identified or, alternatively, some AsmA-like proteins could have intrinsic insertase activity, as it has been recently demonstrated for lipoprotein insertion by LolA in Gram-negative bacteria lacking the OM component LolB of the Lol system (61). Regarding the pathway(s) and mechanism(s) responsible for GPL extraction from the IM, if AsmA-like proteins require a dedicated IM protein machinery for GPL extraction, this should be essential for growth. However, it is hard to believe that it has not yet been identified after decades of research on cell envelope biogenesis in Gram-negative bacteria, unless redundant systems exist (as for AsmA-like proteins themselves) or protein machineries responsible for other essential transport processes are involved. The most obvious candidates could be the LptBFG or the LolCDE complexes, responsible for ATP-dependent extraction from the IM of LPS and lipoproteins, respectively (14–17). However, the first amino acids of mature OM lipoproteins appear to play a prominent role in substrate binding by LolCDE (62), and biochemical and structural studies strongly suggest that LptBFG specifically binds LPS (63–65). Thus, unless their specificity is somehow affected by AsmA-like proteins or other still unidentified modulators, these ABC transporters are unlikely to account for GPL extraction from the IM and transfer to the AsmA-like proteins. Alternatively, the GPL flux toward the periplasmic groove of AsmA-like proteins could be directly driven by GPL synthesis at the IM, a mechanism that would not require the involvement of a specific energy-dependent transporter and that has been hypothesized for eukaryotic inter-organelle GPL transporters (27, 66). The high-flux diffusive flow of GPLs from the IM to the OM that has been observed in *E. coli* (24) would however require the presence of spots of very intensive GPL synthesis or local lipid packing perturbations that could facilitate an efficient loading of AsmA-like proteins with GPLs. In the coming years, we expect much exciting research to clarify these issues and finally unravel the mechanism of anterograde GPL transport in Gram-negative bacteria.

## MATERIALS AND METHODS

### Bacterial strains, plasmids and growth conditions

Strains and plasmids used in this study are listed in Tables S1 and S2, respectively. Bacteria were routinely cultured in Lysogeny broth, Lennox formulation (LB) for genetic manipulation. MH broth was used for plate efficiency and growth assays, permeability assays, and confocal microscopy studies. When required, antibiotics were added at the following concentration for *E. coli* (the concentrations used for *P. aeruginosa* are shown in brackets): ampicillin, 100 µg/mL; tetracycline, 12.5 µg/mL (50–100 μg/mL); carbenicillin (500 µg/mL); nalidixic acid, 15 µg/mL; and chloramphenicol, 30 µg/mL (375 µg/mL).

### Generation of plasmids and recombinant strains

To obtain the constructs for the in-frame deletion of the *P. aeruginosa tamB*, *yhdP*, *yhjG*, *asmA*, *ydbH*, PA4735, and PA2708 genes, two DNA fragments of approximately 500 bp each, encompassing the upstream and downstream region of each gene of interest, were PCR amplified, directionally cloned into the *sacB*-based suicide vector pDM4 (67), and verified by DNA sequencing. Primers and restriction enzymes used for PCR and cloning are listed in Table S4. The resulting pDM4 derivatives were transferred into *P. aeruginosa* by conjugation, and transconjugants were selected on LB agar plates containing 15 µg/mL nalidixic acid and 375 µg/mL chloramphenicol. Deletion mutations were obtained by recombination and sucrose-based selection as previously described (68). Gene deletions were verified by PCR. To generate the *tamB* and the *lptB* conditional mutants, the *tamB* and the *lptB* coding sequences were individually PCR amplified and directionally cloned into the mini-CTX1 derivative pJM253 (69) downstream of the *rhaSR*-P_rhaBAD_ regulatory element. The resulting pJM253 derivatives were transferred into *P. aeruginosa* by conjugation, and transconjugants were selected on LB agar plates containing 15 µg/mL nalidixic acid and 100 µg/mL tetracycline. The plasmid backbone was removed using the *sacB*-based suicide vector pFLP2 (70) as previously described (68), and pFLP2 was cured by plating onto LB agar plates supplemented with 10% sucrose. Carbenicillin-sensitive clones were analyzed by colony PCR to verify the insertion of the *rhaSR*-P_rhaBAD_::*tamB* or *rhaSR*-P_rhaBAD_::*lptB* construct into the chromosome. Then, in-frame deletion of the endogenous copies of *tamB* or *lptB* was obtained by recombination and sucrose-based selection using the pDM4*tamB* and pDM4*lptB* constructs under permissive condition (i.e. growth in the presence of 0.01% rhamnose). The complementing plasmids pME*yhdP*, pME*ydbH*, and pMEPA4735 (Table S2) were generated by individually cloning the PCR-amplified coding sequence of *yhdP*, *ydbH*, or PA4735 into the shuttle vector pME6032, under the control of an IPTG-inducible promoter (71). All constructs were verified by DNA sequencing.

### Plating efficiency and growth assays

For plating efficiency assays, bacterial strains were precultured in MH, supplemented with 0.01% rhamnose for the conditional mutants, harvested by centrifugation, and resuspended in sterile saline solution at OD_600_ = 1. Serial 10-fold dilutions were prepared, and 5-µL aliquots of selected dilutions were spotted onto MH agar plates containing or not 0.25% SDS and 0.25 mM EDTA, 100 or 1,000 µg/mL vancomycin (corresponding to 0.05× or 0.5× MIC for the PAO1 wild-type strain, respectively), or 0.125 or 0.25 µg/mL colistin (corresponding to 0.25× and 0.5× MIC for the PAO1 wild-type strain, respectively) in the presence or not of 0.01% rhamnose or 0.5 mM IPTG, when appropriate.

For planktonic growth assays, bacterial strains were precultured in MH, supplemented with 0.01% rhamnose for the conditional mutants, and then refreshed 1:1,000 in MH in the absence or presence of 0.01% rhamnose, 0.5 mM IPTG, or increasing concentrations of the LpxC inhibitor PF-5081090 (43), when appropriate. Bacterial cultures were incubated in 96-well microtiter plates (200 µL in each well) at 37°C in a Tecan Spark 10M microtiter plate reader, and growth was measured over time as the OD_600_ of the bacterial cultures.

TamB-depleted cells of the conditional mutant Δ*yhdP* Δ*ydbH* Δ*tamB rhaSR*-P*_rhaBAD_::tamB* ΔPA4735 (Table S1), which were used for microscopy and cell envelope stability assays, were obtained through a previously described dual-refresh culturing strategy (72). Briefly, cells were cultured overnight in the presence of 0.01% rhamnose and then refreshed at high cell density (1:30 dilution) in the absence of rhamnose, cultured for 2 h, and then refreshed again (1:50 dilution) in the same medium. Cells were collected as soon as a growth defect was observed in the conditional mutant with respect to the wild-type strain (Fig. S4).

### Confocal microscopy

Bacterial strains carrying the constructs pME*ftsZ-GFP* were cultured in flask in MH, supplemented or not with 0.01% rhamnose, in the presence of 0.003 mM IPTG to induce non-toxic expression levels of the FtsZ-GFP fusion protein (35). Two hundred microliters of late-exponential phase cultures were collected, the fluorescent dye FM4-64 (73) was added at 5 µg/mL, and the samples were incubated at 37°C for 30 min. Then, 5 µL of the bacterial cell suspensions were spotted on a microscope glass slide overlaid with 0.5% agarose and imaged with a Nikon A1R HD25 confocal laser scanning microscope equipped with an Apo TIRF 100× oil immersion objective (NA 1.49). The 488- and 561-nm laser lines were employed for the GFP and FM4-64 excitation, respectively. Emission bandwidths at 500–540 nm and 600–720 nm were used for GFP and FM4-64 detection, respectively. Images were acquired at a sampling dimension of 512 × 512 pixels and were deconvoluted using the NIS-Elements software (Nikon), using default parameters. Image processing to determine cell morphological parameters was performed according to a previously established pipeline (74). Cell area and aspect ratio were calculated using ImageJ v.1.53c for 100 cells of each bacterial strain (75).

### Membrane permeability assays

Bacterial strains were cultured in MH, supplemented or not with 0.01% rhamnose, in the absence or presence of the LpxC inhibitor PF-5081090 at 0.031× MIC (corresponding to 0.008 µg/mL for PAO1 and 0.004 µg/mL for Δ*tamB* Δ*yhdP* Δ*ydbH*). Exponential phase cells were harvested by centrifugation and resuspended in 5 mM HEPES (pH 7.2) at OD_600_ = 3. Equal volumes (150 µL) of HEPES solution containing or not PI (40 µg/ml) or NPN (20 µM) and the bacterial suspensions were mixed, and 100 µL of each sample was aliquoted on a black flat-bottom 96-well plate. OD_600_ and fluorescence were measured in a Tecan Spark 10M microtiter plate reader (excitation at 350 nm and emission at 420 nm for NPN; excitation at 580 nm and emission at 620 nm for PI) after 2 min at room temperature, subtracted of the background values of samples without NPN or PI, and normalized to the OD_600_ of the cell suspension (76).

### MIC assays

The MIC of antibiotics (vancomycin and colistin) or the LpxC inhibitor PF-5081090 were determined through the broth microdilution method. *P. aeruginosa* strains precultured in MH were refreshed in the same medium at ca. 5 × 10^5^ cells/mL in the presence of increasing concentrations of each antibiotic/compound. MIC was recorded after 20 h at 37°C. At least three independent experiments were performed for each strain/compound.

### Bioinformatic analyses

TMHs were predicted using the online tool TMHMM 2.0 (https://services.healthtech.dtu.dk/services/TMHMM-2.0/) (77). Predicted 3D structures were retrieved from the AlphaFold Protein Structure Database (https://alphafold.ebi.ac.uk/) (28). Orthologs within the *Pseudomonas* genus were identified using the Ortholog Group Members function of the *Pseudomonas* Genome Database (https://pseudomonas.com/) (78). Putative structural homologs of PA4735 were searched with Foldseek (https://search.foldseek.com/search) (79), by using the PDB downloaded by the AlphaFold Database as query.

### Statistical analysis

Statistical analysis was performed with the software GraphPad Instat, using the unpaired t test or the ANOVA test as indicated.

## SUPPLEMENTAL MATERIAL

Supplemental figures and tables msphere.00677-23-s0001.pdf Tables S1 to S4 and Figures S1 to S5.

ASM does not own the copyrights to Supplemental Material that may be linked to, or accessed through, an article. The authors have granted ASM a non-exclusive, world-wide license to publish the Supplemental Material files. Please contact the corresponding author directly for reuse.
