# Supplementary material for: Redundant essentiality of AsmA-like proteins in Pseudomonas aeruginosa
Source: mSphere. 2024 Feb 2;9(2):e00677-23. doi: 10.1128/msphere.00677-23 (PMC10900882; doi:10.1128/msphere.00677-23)
Supplement: Supplemental figures and tables — Tables S1 to S4 and Figures S1 to S5. [file msphere.00677-23-s0001.pdf]

Redundant essentiality of AsmA-like proteins in *Pseudomonas aeruginosa*

Davide Sposato, Jessica Mercolino, Luisa Torrini, Paola Sperandeo, Massimiliano Lucidi, Riccardo Alegiani, Ilaria Varone, Giorgia Molesini, Livia Leoni, Giordano Rampioni, Paolo Visca, Francesco Imperi

SUPPLEMENTARY MATERIAL

Table S1. Bacterial strains used in this study.

Table S2. Plasmids used in this study.

Table S3. Putative structural homologs of PA4735 identified by FoldSeek.

Table S4. Primers used to generate the plasmids described in Table S2.

Figure S1. Plating efficiency of the *P. aeruginosa* mutants with single, double or triple deletions in *asmA*-like genes that showed defects with respect to the wild type strain.

Figure S2. Structural alignment of PA4735 with selected putative structural homologs.

Figure S3. Confocal microscopy analysis of the single mutants Δ*tamB*, Δ*yhdP*, Δ*ydbH*, and ΔPA4735.

Figure S4. Culturing strategy used to obtain *P. aeruginosa* cells depleted in AsmA-like proteins of interest.

Figure S5. Confocal microscopy images of selected *P. aeruginosa* mutants in *asmA*-like genes expressing a FtsZ-GFP fusion protein.

Table S1. Bacterial strains used in this study.

Strain	Genotype or relevant characteristics	Source or reference
E. coli		
S17.1λ*pir*	*thi pro hsdR hsdM*$^{+}$ *recA* RP4-2-Tc::Mu-Km::Tn7λ*pir,* Gm^{R}	Simon *et al.*, 1983[a]
P. aeruginosa		
PAO1 (ATCC15692)	Reference isolate, wild type	American Type Culture Collection
Δ*tamA*	PAO1 with an in-frame deletion in the *tamA* (PA2543) coding sequence	This study
Δ*tamB*	PAO1 with an in-frame deletion in the *tamB* (PA2542) coding sequence	This study
Δ*asmA*	PAO1 with an in-frame deletion in the *asmA* (PA5146) coding sequence	This study
Δ*yhdP*	PAO1 with an in-frame deletion in the *yhdP* (PA4476) coding sequence	This study
Δ*yhjG*	PAO1 with an in-frame deletion in the *yhjG* (PA4879) coding sequence	This study
Δ*ydbH*	PAO1 with an in-frame deletion in the *ydbH* (PA5307) coding sequence	This study
ΔPA4735	PAO1 with an in-frame deletion in the PA4735 coding sequence	This study
ΔPA2708	PAO1 with an in-frame deletion in the PA2708 coding sequence	This study
Δ*tamB* Δ*asmA*	Δ*tamB* deleted of *asmA*	This study
Δ*tamB* Δ*yhdP*	Δ*tamB* deleted of *yhdP*	This study
Δ*tamB* Δ*yhjG*	Δ*tamB* deleted of *yhjG*	This study
Δ*asmA* Δ*yhdP*	Δ*asmA* deleted of *yhdP*	This study
Δ*asmA* Δ*yhjG*	Δ*asmA* deleted of *yhjG*	This study
Δ*yhdP* Δ*yhjG*	Δ*yhdP* deleted of *yhjG*	This study
Δ*yhjG* Δ*ydbH*	Δ*yhjG* deleted of *ydbH*	This study
Δ*tamB* Δ*ydbH*	Δ*tamB* deleted of *ydbH*	This study
Δ*asmA* Δ*ydbH*	Δ*asmA* deleted of *ydbH*	This study
Δ*yhdP* Δ*ydbH*	Δ*yhdP* deleted of *ydbH*	This study
Δ*tamB* ΔPA4735	Δ*tamB* deleted of PA4735	This study
Δ*asmA* ΔPA4735	Δ*asmA* deleted of PA4735	This study
Δ*yhdP* ΔPA4735	Δ*yhdP* deleted of PA4735	This study
Δ*yhjG* ΔPA4735	Δ*yhjG* deleted of PA4735	This study
Δ*ydbH* ΔPA4735	Δ*ydbH* deleted of PA4735	This study
Δ*tamB* Δ*yhdP* Δ*ydbH*	Δ*tamB* Δ*yhdP* deleted of *ydbH*	This study
Δ*yhdP* Δ*ydbH* Δ*tamA*	Δ*yhdP* Δ*ydbH* deleted of *tamA*	This study
Δ*tamB* Δ*asmA* Δ*yhdP*	Δ*tamB* Δ*asmA* deleted of *yhdP*	This study
Δ*tamB* Δ*asmA* Δ*yhjG*	Δ*tamB* Δ*asmA* deleted of *yhjG*	This study
Δ*tamB* Δ*yhdP* Δ*yhjG*	Δ*tamB* Δ*yhdP* deleted of *yhjG*	This study
Δ*asmA* Δ*yhdP* Δ*yhjG*	Δ*asmA* Δ*yhdP* deleted of *yhjG*	This study
Δ*tamB* Δ*asmA* Δ*ydbH*	Δ*tamB* Δ*asmA* deleted *ydbH*	This study
Δ*tamB* Δ*yhjG* Δ*ydbH*	Δ*tamB* Δ*yhjG* deleted of *ydbH*	This study
Δ*asmA* Δ*yhdP* Δ*ydbH*	Δ*asmA* Δ*yhdP* deleted of *ydbH*	This study
Δ*asmA* Δ*yhjG* Δ*ydbH*	Δ*asmA* Δ*yhjG* deleted of *ydbH*	This study
Δ*yhdP* Δ*yhjG* Δ*ydbH*	Δ*yhdP* Δ*yhjG* deleted of *ydbH*	This study
Δ*tamB* Δ*asmA* ΔPA4735	Δ*tamB* Δ*asmA* deleted of PA4735	This study
Δ*tamB* Δ*yhdP* ΔPA4735	Δ*tamB* Δ*yhdP* deleted of PA4735	This study
Δ*tamB* Δ*yhjG* ΔPA4735	Δ*tamB* Δ*yhjG* deleted of PA4735	This study
Δ*asmA* Δ*yhdP* ΔPA4735	Δ*asmA* Δ*yhdP* deleted of PA4735	This study
Δ*asmA* Δ*yhjG* ΔPA4735	Δ*asmA* Δ*yhjG* deleted of PA4735	This study
Δ*yhdP* Δ*yhjG* ΔPA4735	Δ*yhdP* Δ*yhjG* deleted of PA4735	This study
Δ*yhjG* Δ*ydbH* ΔPA4735	Δ*yhjG* Δ*ydbH* deleted of PA4735	This study
Δ*tamB* Δ*ydbH* ΔPA4735	Δ*tamB* Δ*ydbH* deleted of PA4735	This study
Δ*asmA* Δ*ydbH* ΔPA4735	Δ*asmA* Δ*ydbH* deleted of PA4735	This study

Δ*yhdP* Δ*ydbH* ΔPA4735	Δ*yhdP* Δ*ydbH* deleted of PA4735	This study
Δ*yhdP* Δ*ydbH* *rhaSR*-P$_{rhaBAD}$::*tamB*	Δ*yhdP* Δ*ydbH* with a rhamnose-inducible additional copy of the *tamB* coding sequence inserted into the *attB* neutral site	This study
Δ*yhdP* Δ*ydbH* Δ*tamB* *rhaSR*-P$_{rhaBAD}$::*tamB*	Δ*yhdP* Δ*ydbH* *rhaSR*-P$_{rhaBAD}$::*tamB* with an in-frame deletion in the endogenous copy of *tamB*	This study
Δ*yhdP* Δ*ydbH* Δ*tamB* *rhaSR*-P$_{rhaBAD}$::*tamB* Δ*asmA*	Δ*yhdP* Δ*ydbH* Δ*tamB* *rhaSR*-P$_{rhaBAD}$::*tamB* deleted of *asmA*	This study
Δ*yhdP* Δ*ydbH* Δ*tamB* *rhaSR*-P$_{rhaBAD}$::*tamB* Δ*yhjG*	Δ*yhdP* Δ*ydbH* Δ*tamB* *rhaSR*-P$_{rhaBAD}$::*tamB* deleted of *yhjG*	This study
Δ*yhdP* Δ*ydbH* Δ*tamB* *rhaSR*-P$_{rhaBAD}$::*tamB* ΔPA4735	Δ*yhdP* Δ*ydbH* Δ*tamB* *rhaSR*-P$_{rhaBAD}$::*tamB* deleted of PA4735	This study
Δ*yhdP* Δ*ydbH* Δ*tamB* *rhaSR*-P$_{rhaBAD}$::*tamB* ΔPA2708	Δ*yhdP* Δ*ydbH* Δ*tamB* *rhaSR*-P$_{rhaBAD}$::*tamB* deleted of PA2708	This study
rhaSR-P$_{rhaBAD}$::*lptB*	PAO1 with a rhamnose-inducible additional copy of the *lptB* (PA4461) coding sequence inserted into the *attB* neutral site	This study
Δ*lptB* *rhaSR*-P$_{rhaBAD}$::*lptB*	*rhaSR*-P$_{rhaBAD}$::*lptB* with an in-frame deletion in the endogenous copy of *lptB*	This study
Δ*tamB* Δ*yhdP* Δ*ydbH* *rhaSR*-P$_{rhaBAD}$::*lptB*	Δ*tamB* Δ*yhdP* Δ*ydbH* with a rhamnose-inducible additional copy of the *lptB* (PA4461) coding sequence inserted into the *attB* neutral site	This study
Δ*tamB* Δ*yhdP* Δ*ydbH* Δ*lptB* *rhaSR*-P$_{rhaBAD}$::*lptB*	Δ*tamB* Δ*yhdP* Δ*ydbH* *rhaSR*-P$_{rhaBAD}$::*lptB* with an in-frame deletion in the endogenous copy of *lptB*	This study

[1] Reference not included in the main text: Simon R, Priefer U, Pühler A. A Broad Host Range Mobilization System for In Vivo Genetic Engineering: Transposon Mutagenesis in Gram Negative Bacteria. Nat Biotechnol 1983; 1:784-791. doi.org/10.1038/nbt1183-784.

Table S2. Plasmids used in this study.

Plasmid	Relevant characteristics	Source or reference
pDM4	Suicide vector in *P. aeruginosa*; *sacB*, *oriR6K*; Cm^R	67
pDM4Δ*tamB*	pDM4 derivative carrying the DNA regions upstream and downstream of the *tamB* coding sequence, used for the in-frame deletion of *tamB* by homologous recombination	This study
pDM4Δ*tamA*	pDM4 derivative carrying the DNA regions upstream and downstream of the *tamA* coding sequence, used for the in-frame deletion of *tamA* by homologous recombination	This study
pDM4Δ*yhdP*	pDM4 derivative carrying the DNA regions upstream and downstream of the *yhdP* coding sequence, used for the in-frame deletion of *yhdP* by homologous recombination	This study
pDM4Δ*asmA*	pDM4 derivative carrying the DNA regions upstream and downstream of the *asmA* coding sequence, used for the in-frame deletion of *asmA* by homologous recombination	This study
pDM4Δ*ydbH*	pDM4 derivative carrying the DNA regions upstream and downstream of the *ydbH* coding sequence, used for the in-frame deletion of *ydbH* by homologous recombination	This study
pDM4Δ*yhjG*	pDM4 derivative carrying the DNA regions upstream and downstream of the *yhjG* coding sequence, used for the in-frame deletion of *yhjG* by homologous recombination	This study
pDM4ΔPA4735	pDM4 derivative carrying the DNA regions upstream and downstream of the PA4735 coding sequence, used for the in-frame deletion of PA4735 by homologous recombination	This study
pDM4ΔPA2708	pDM4 derivative carrying the DNA regions upstream and downstream of the PA2708 coding sequence, used for the in-frame deletion of PA2708 by homologous recombination	This study
pDM4Δ*lptB*	pDM4 derivative carrying the DNA regions upstream and downstream of the *lptB* coding sequence, used for the in-frame deletion of *lptB* by homologous recombination	This study
pJM253	mini-CTX1 derivative carrying *rhaRS*-P_{rhaBAD}, Tc^R	69
pJM253*tamB*	pJM253 derivative carrying the coding sequence of *tamB* downstream of P_{rhaBAD}	This work
pJM253*lptB*	pJM253 derivative carrying the coding sequence of *lptB* downstream of P_{rhaBAD}	This work
pME6032	IPTG inducible expression vector, *lacI*^{q}-P_{tac}, Tc^R	71
pME*ftsZ-GFP*	pME6032 derivative carrying the coding sequence of *ftsZ* fused to the coding sequence of the GFP gene downstream of the IPTG-inducible P_{tac} promoter	35
pME*yhdP*	pME6032 derivative carrying the coding sequence of *yhdP* downstream of the IPTG-inducible P_{tac} promoter	This work
pME*ydbH*	pME6032 derivative carrying the coding sequence of *ydbH* downstream of the IPTG-inducible P_{tac} promoter	This work
pMEPA4735	pME6032 derivative carrying the coding sequence of PA4735 downstream of the IPTG-inducible P_{tac} promoter	This work
pFLP2	Broad-host-range plasmid expressing the Flp recombinase, *sacB*; Ap^R/Cb^R	70

Table S3. Putative structural homologs of PA4735 identified by FoldSeek in the AlphaFold database.[a]

Target	Species	Phylum (class)	Gene ID	Description	Sequence identity	E-Value	Score	Query position	Target position
AF-A0A4R2F9D2-F1-model_v4	*Shewanella fodinae*	Pseudomonadota (Gammaproteobacteria)	EDC91_11419	Uncharacterized protein DUF748	26	2.05e-89	3265	1-1088 (1088)	16-1021 (1021)
AF-B1ZZF4-F1-model_v4	*Opitutus terrae*	Verrucomicrobiota (Opitutae)	Oter_3077	Uncharacterized protein	23.9	2.86e-88	3203	5-1086 (1088)	6-1041 (1041)
AF-A0A2U8E1C4-F1-model_v4	*Ereboglobus luteus*	Verrucomicrobiota (Opitutae)	CKA38_04335	Uncharacterized protein	21.9	1.66e-84	3175	1-1086 (1088)	22-1002 (1002)
AF-B1ZQ83-F1-model_v4	*Opitutus terrae*	Verrucomicrobiota (Opitutae)	Oter_0273	Uncharacterized protein	20.2	2.21e-81	2908	1-1086 (1088)	2-1098 (1098)
AF-A0A443XFD8-F1-model_v4	*Aeromonas caviae*	Pseudomonadota (Gammaproteobacteria)	DN604_14145	Uncharacterized protein	26	9.01e-65	2510	235-1087 (1088)	5-718 (718)
AF-A0A2G6MLV2-F1-model_v4	*Desulfobacterales* bacterium	Thermodesulfobacteriota (Desulfobacteria)	CSA29_06130	Uncharacterized protein	18.5	4.63e-78	2398	1-1086 (1088)	1-1232 (1232)
AF-A0A496LTU2-F1-model_v4	*Campylobacter* sp.	Campylobacterota (Epsilonproteobacteria)	D8H92_03425	DUF748 domain-containing protein	17.3	2.02e-75	2102	3-1086 (1088)	25-1137 (1138)
AF-A0A2V6PN49-F1-model_v4	Candidatus *Rokubacteria* bacterium	Candidate phylum Rokubacteria	DMD81_19255	Uncharacterized protein	13.8	2.00e-59	1782	1-1087 (1088)	1-1102 (1132)
AF-A0A1Q6DQI5-F1-model_v4	*Desulfobulbaceae* bacterium DB1	Thermodesulfobacteriota (Desulfobulbia)	BM485_04950	Uncharacterized protein	15.6	1.95e-60	1757	9-1087 (1088)	2-1122 (1127)
AF-A0A356VEA3-F1-model_v4	*Halieaceae* bacterium	Pseudomonadota (Gammaproteobacteria)	DD808_03825	Uncharacterized protein	27.3	4.84e-40	1510	572-1088 (1088)	7-509 (509)

[a] Only the 10 putative structural homologs with the highest score are described in the table. The other putative structural homologs can be retrieved at https://search.foldseek.com/result/hPOmiVVMLzrWTF9BD4SI1jJlx0OMQQvtG2jcjw/0.

Table S4. Primers used to generate the plasmids described in Table S2.[a]

Primer	Sequence (5'-3')[1]	Restriction sites[2]	Application
*tamA*_mut_UP_FW	gctctaGACCCGGCGCTACCTGG	XbaI	
*tamA*_mut_UP_RV	cgggatCCGTACTCAGGCACAGCAG	BamHI	Generation of
*tamA*_mut_DOWN_FW	cgggatcCGCTCGACGACGACGGC	BamHI	pDM4Δ*tamA*
*tamA*_mut_DOWN_RV	ccccctCGAGTCGATGTGCAGGCCG	XhoI	
*tamB*_mut_UP_FW	gctctaGACGCCATGGCCAAGGGC	XbaI	
*tamB*_mut_UP_RV	cgggaTCCCAGGAGCAACGCGAG	BamHI	Generation of
*tamB*_mut_DOWN_FW	cgggatCCAAACGCCTCTATCTCGAAG	BamHI	pDM4Δ*tamB*
*tamB*_mut_DOWN_RV	ccccctcgAGGTACAGGCCGCTGTCG	XhoI	
*tamB*_pJM253_FW	ggactagTTCTCCATGGGGCCAGAAC	SpeI	Generation of
*tamB*_pJM253_RV	cccaagcTTACGCATGGTCCGTATCAG	HindIII	pJM253*lptB*
*asmA*_mut_UP_FW	ccgctcgaGCTCGCCGTTCCGCCAC	XhoI	
*asmA*_mut_UP_RV	cgggaTCCCAGGAAGAACAGCCCC	BamHI	Generation of
*asmA*_mut_DOWN_FW	cgggatCCGGCAATCGCCTCAACG	BamHI	pDM4Δ*asmA*
*asmA*_mut_DOWN_RV	gctctaGAGGATCGGTGCGCGCAG	XbaI	
*yhjG*_mut_UP_FW	ccgctcgAGCGCAGGAAGCAGACCAA	XhoI	
*yhjG*_mut_UP_RV	cggaaTTCGGCCGCGCGTCATGC	EcoRI	Generation of
*yhjG*_mut_DOWN_FW	cggaatTCTCGATGTCACCCCGCAC	EcoRI	pDM4Δ*yhjG*
*yhjG*_mut_DOWN_RV	gctctaGAGGGCTGGCCGGTGCC	Xba	
*yhdP*_mut_UP_FW	acgcgtcgACCCTGGAGAAGCAACTGG	SalI	
*yhdP*_mut_UP_RV	cgggatCCAGCAGCACCAGTACCAG	BamHI	Generation of
*yhdP*_mut_DOWN_FW	cgGGATCCGAAGATCAGCTTCG	BamHI	pDM4Δ*yhdP*
*yhdP*_mut_DOWN_RV	gctctaGACCGAAAGCATAGTCGTCG	XbaI	
*ydbH*_mut_UP_FW	ccgctcgAGTCGAAGTGGCCGGACAC	XhoI	
*ydbH*_mut_UP_RV	cgggatCCAGCAGCAACGTCGTCC	BamHI	Generation of
*ydbH*_mut_DOWN_FW	cgggatCCCGGCTACCTGCGCTTC	BamHI	pDM4Δ*ydbH*
*ydbH*_mut_DOWN_RV	gctcTAGAACAGGCCGCTGTTCTC	XbaI	
PA4735_mut_UP_FW	ccgctCGAGCAGGACCGTTACTAC	XhoI	
PA4735_mut_UP_RV	cgggaTCCTTTGGGCATGGCTCTC	BamHI	Generation of
PA4735_mut_DOWN_FW	cgggatCCGAAGGCGAAGACAAGGG	BamHI	pDM4ΔPA4735
PA4735_mut_DOWN_RV	gctctaGACCTCGCGATAACCCAGG	XbaI	
PA2708_mut_UP_FW	ccgctcgAGGACCAGCTCCGCGCC	XhoI	
PA2708_mut_UP_RV	cgggatCCGCCAGGCTGCCCAGG	BamHI	Generation of
PA2708_mut_DOWN_FW	cgggatCCGGCGACGCGCGACG	BamHI	pDM4ΔPA2708
PA2708_mut_DOWN_RV	gctctAGAGCTTGCCCTTCTTGATG	XbaI	
*lptB*_mut_UP_FW	ccgctcgaGAACAACCGATCCGCGTCC	XhoI	
*lptB*_mut_UP_RV	cgggatCCAGGTGCTGGGCTTTGAG	BamHI	Generation of
*lptB*_mut_DOWN_FW	cgggatccGGTCAAGGAAGTCTACCTGG	BamHI	pDM4Δ*lptB*
*lptB*_mut_DOWN_RV	gctctaGAGGCTTTCGACGCTGGG	XbaI	
*lptB*_pJM253_FW	ggactaGTCATCCAGCCGAAGAAAAAG	SpeI	Generation of
*lptB*_pJM253_RV	cccaagcTTCTTCAGAGGCGGAACTCG	HindIII	pJM253*lptB*
*yhdP*_pME6032_FW	cccgagctcATGGCGGATGTGGGAAGCC	SacI	Generation of
*yhdP*_pME6032_RV	ccccctcgaGACATGGTTTCCTCCTTGCG	XhoI	pME*yhdP*
*ydbH*_pME6032_FW	gccgagctCATGCGTTTGCGCAGAAGG	SacI	Generation of
*ydbH*_pME6032_RV	catgccatGGCGCATCGGGGGTTCTC	NcoI	pME*ydbH*
PA4735_pME6032_FW	ggcgagctCATGCCCAAAGGACTGAAA C	SacI	Generation of pMEPA4735
PA4735_pME6032_RV	catgccatGGCGAATCGCTTGTGCAAAG	NcoI	

[a] Preparative PCRs for cloning were performed using the genomic DNA of *P. aeruginosa* PAO1 as the template.

[b] The restriction site used for cloning is underlined in the primer sequence.

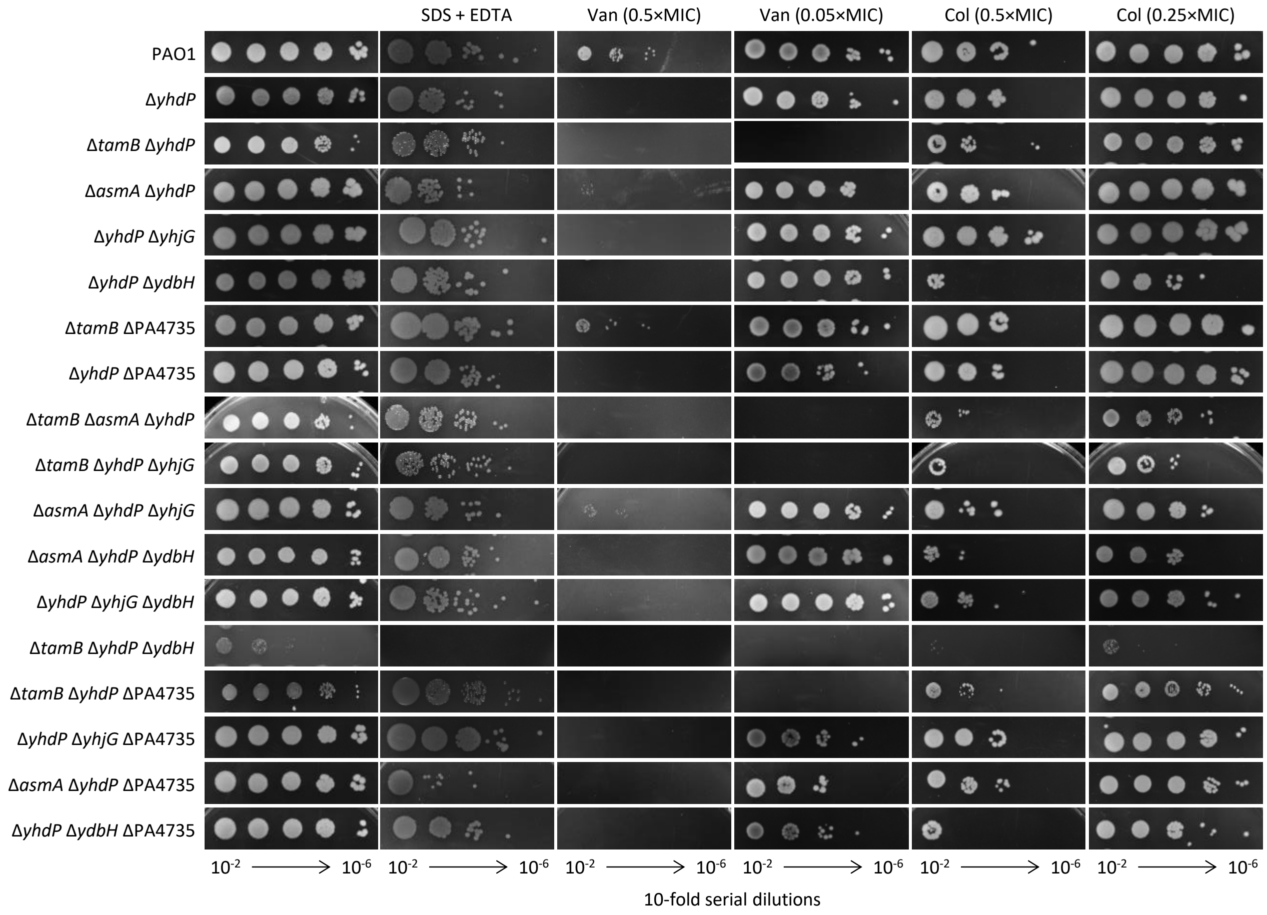

Figure S1. Plating efficiency of the *P. aeruginosa* wild type PAO1 and all isogenic mutants with single, double or triple deletions in *asmA*-like genes that showed defects with respect to the wild type strain on MH agar plates supplemented or not with SDS and EDTA (0.25% and 0.25 mM, respectively), vancomycin (Van) or colistin (Col) at concentrations corresponding to 0.05, 0.25 or 0.5×MIC for the wild type strain (as indicated). Images were taken after 24 h of incubation at 37°C and are representative of at least three independent experiments. All the other mutants generated in this work (Table S1) and not included in this figure did not show plating efficiency defects under the conditions tested (data not shown).

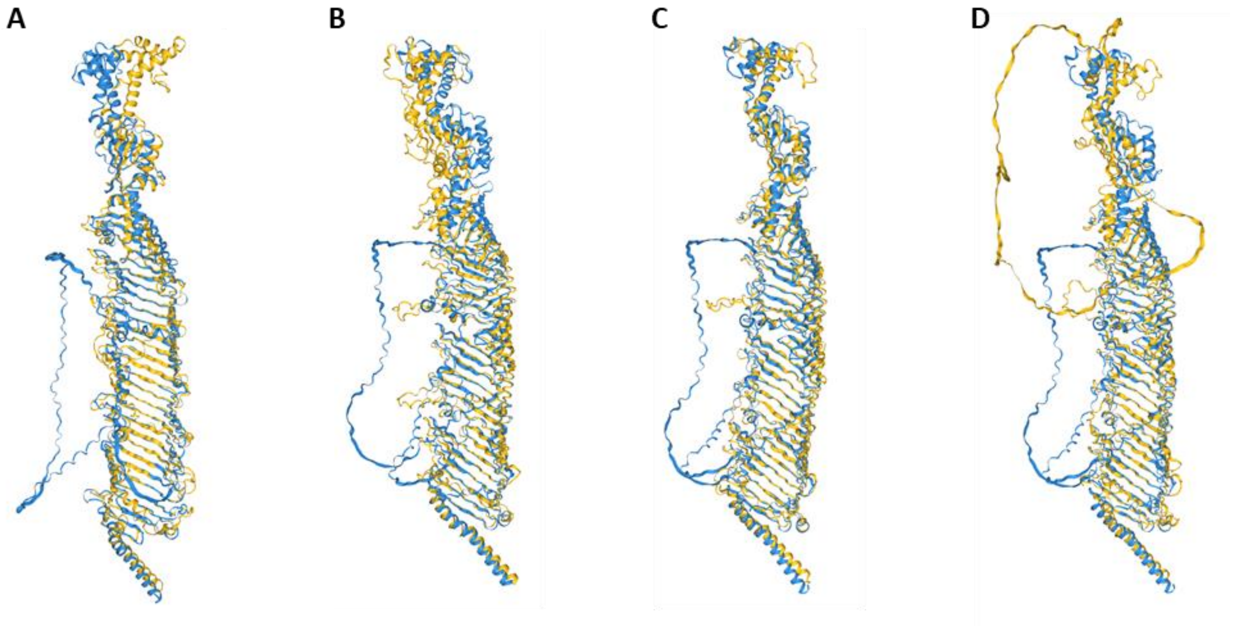

Figure S2. Structural alignment of PA4735 with the proteins encoded by (**A**) the EDC91_11419 gene of *Shewanella fodinae*, (**B**) the Oter_3077 gene of *Opitutus terrae*, (**C**) the CKA38_04335 gene of *Ereboglobus luteus*, and (**D**) the Oter_0273 gene of *Opitutus terrae*, predicted by FoldSeek (Table S3). The PA4735 protein is in blue, while the putative PA4735 structural homologs are in yellow.

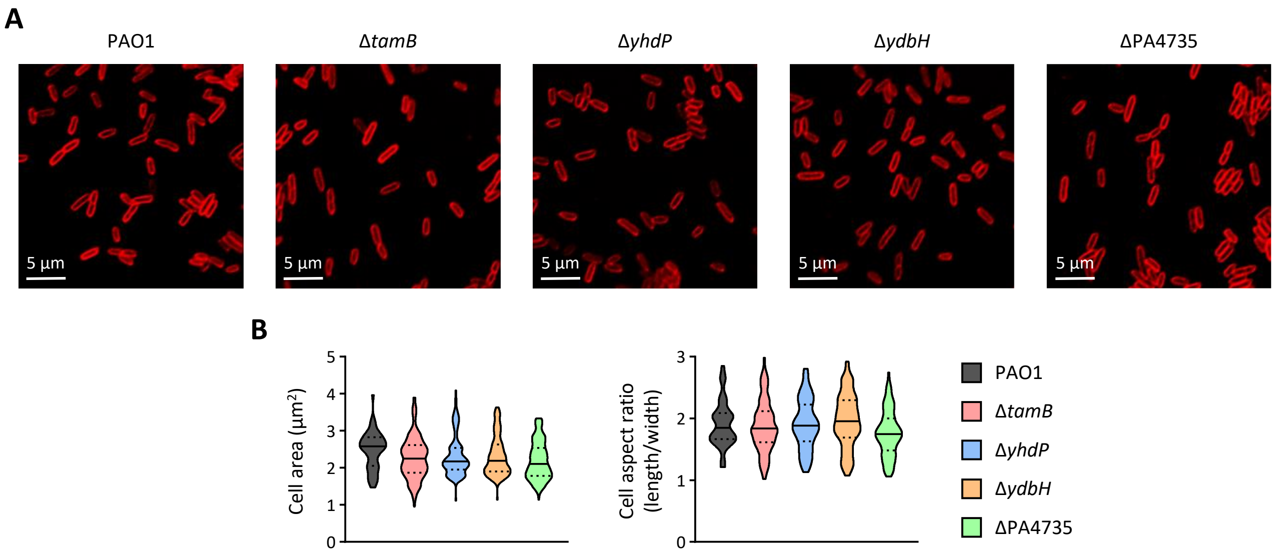

Figure S3. (A) Confocal microscopy images of *P. aeruginosa* PAO1 and the single deletion mutants in *tamB*, *yhdP*, *ydbH* or PA4735 cultured in MH until the mid-exponential phase and stained with the membrane-labelling dye FM4-64. Images are representative of two independent experiments and several fields of view showing the same results. (B) Violin plots showing cell area (μm^2) and aspect ratio (length/width) for 100 cells of the same strains shown in panel A, calculated with the ImageJ software.

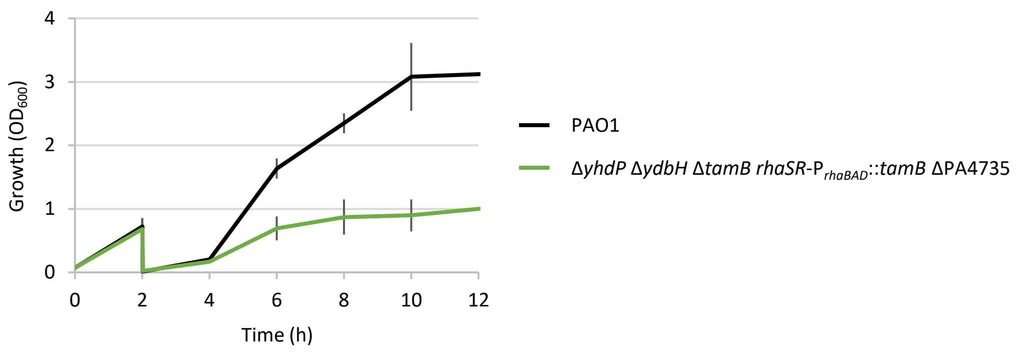

Figure S4. Dual-refresh culturing strategy used to obtain TamB-depleted cells of the conditional mutant Δ*yhdP* Δ*ydbH* Δ*tamB rhaSR*-P*rhaBAD*::*tamB* ΔPA4735. *P. aeruginosa* PAO1 and the conditional mutant were cultured overnight in flasks at 200 rpm and 37°C in MH broth supplemented with 0.01% rhamnose (not shown) and then diluted 1:30 in MH broth (without rhamnose). After 2 h, the cultures were further diluted 1:50 in fresh medium, and growth (OD_{600}) was monitored over time. Values are the mean (± standard deviation) of three independent experiments. Bacterial cells were collected for microscopy and cell envelope stability assays as soon as a growth defect was observed in the conditional mutant with respect to the wild type strain (6 h).

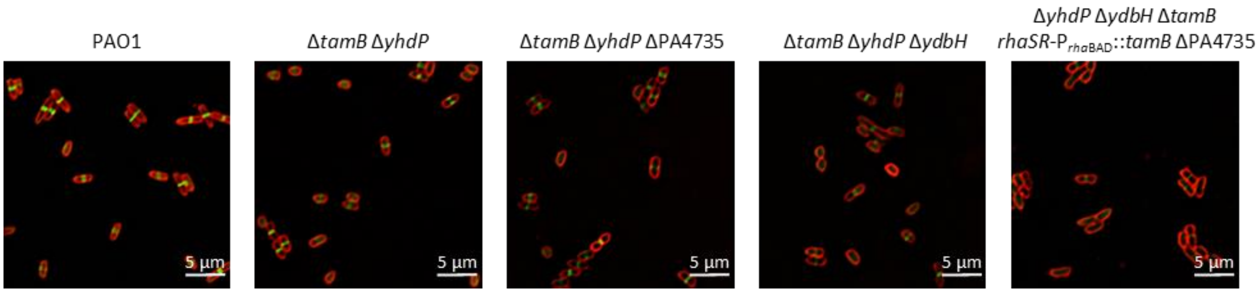

Figure S5. Confocal microscopy images of *P. aeruginosa* PAO1 and the mutants Δ*tamB* Δ*yhdP*, Δ*tamB* Δ*yhdP* Δ*ydbH,* Δ*tamB* Δ*yhdP* ΔPA4735, and Δ*yhdP* Δ*ydbH* Δ*tamB* *rhaSR*-P_{rhaBAD}::*tamB* ΔPA4735 carrying the plasmid pME *ftsZ-GFP,* cultured in the presence of 0.003 mM IPTG to induce the expression of the fusion protein FtsZ-GFP at non-toxic levels (35) and stained with the membrane-labelling dye FM4-64. Strains were cultured in MH until the mid-exponential phase, except for the conditional mutant Δ*yhdP* Δ*ydbH* Δ*tamB* *rhaSR*-P_{rhaBAD}::*tamB* ΔPA4735 that was progressively depleted of TamB using the culturing strategy shown in Figure S4. Images are representative of two independent experiments and several fields of view showing the same results.
